# Messenger RNA sequencing and pathway analysis provide novel insights into the biological basis of chickens’ feed efficiency

**DOI:** 10.1186/s12864-015-1364-0

**Published:** 2015-03-17

**Authors:** Nan Zhou, William R Lee, Behnam Abasht

**Affiliations:** Department of Animal and Food Sciences, University of Delaware, Newark, DE 19716, USA; Maple Leaf Farms LLC, Leesburg, IN 46538, USA

**Keywords:** RNA-seq, Differential expression analysis, Chicken feed efficiency, Breast muscle, Muscle remodeling, IGFs/PI3K/Akt signaling pathway

## Abstract

**Background:**

Advanced selection technologies have been developed and continually optimized to improve traits of agricultural importance; however, these methods have been primarily applied without knowledge of underlying biological changes that may be induced by selection. This study aims to characterize the biological basis of differences between chickens with low and high feed efficiency (FE) with a long-term goal of improving the ability to select for FE.

**Results:**

High-throughput RNA sequencing was performed on 23 breast muscle samples from commercial broiler chickens with extremely high (*n* = 10) and low (*n* = 13) FE. An average of 34 million paired-end reads (75 bp) were produced for each sample, 80% of which were properly mapped to the chicken reference genome (Ensembl *Galgal4*). Differential expression analysis identified 1,059 genes (FDR < 0.05) that significantly divergently expressed in breast muscle between the high- and low-FE chickens. Gene function analysis revealed that genes involved in muscle remodeling, inflammatory response and free radical scavenging were mostly up-regulated in the high-FE birds. Additionally, growth hormone and IGFs/PI3K/Akt signaling pathways were enriched in differentially expressed genes, which might contribute to the high breast muscle yield in high-FE birds and partly explain the FE advantage of high-FE chickens.

**Conclusions:**

This study provides novel insights into transcriptional differences in breast muscle between high- and low-FE broiler chickens. Our results show that feed efficiency is associated with breast muscle growth in these birds; furthermore, some physiological changes, e.g., inflammatory response and oxidative stress, may occur in the breast muscle of the high-FE chickens, which may be of concern for continued selection for both of these traits together in modern broiler chickens.

**Electronic supplementary material:**

The online version of this article (doi:10.1186/s12864-015-1364-0) contains supplementary material, which is available to authorized users.

## Background

Genetic selection has tremendously improved livestock and plant production over the past 50 years [1,2]. Advanced selection technologies have been developed and continually optimized to genetically improve traits of agricultural importance [1,3,4]. However, these methods have been primarily applied without knowledge of underlying biological changes that may be induced by selection [5,6]. Previous studies reported possible association between selection for improved performance and increased rate of physiological and metabolic disorders in modern breeds [7-9]. For example, chickens and turkeys selected for high growth rate have shown increased incidence of muscle disorders, heart failure syndrome and ascites [10-12]. A detailed characterization of traits of breeding interest may help to anticipate unfavorable consequences of long-term selection programs and adjust or perhaps redefine breeding objectives accordingly.

One of the most important traits in broiler chicken production is feed efficiency (FE), which defines the chicken’s ability to convert feed into body weight. As feed cost represents nearly 70% of the total cost in poultry production, improving FE has been an important goal in broiler chicken breeding programs [13]. Selection for FE in broiler chickens can be accomplished using different measurements and procedures. A widely used measure of FE in broiler chickens is residual feed consumption (RFC), which is defined as the difference between an animal’s actual feed intake and expected feed intake on the basis of body weight and growth [13]. Although moderate heritability, ranging from 0.42 to 0.45, for RFC has been reported in a previous study using more diverse chicken populations [14], to our knowledge this trait exhibits lower heritability (~0.2) in the commercial pure lines, possibly explaining the relatively slow progress in improving FE in commercial breeding programs. Insights into the biological basis of differences in chicken FE are required to develop more efficient and sustainable selection strategies.

Previous studies have revealed a link between mitochondrial function and FE in broiler chickens. Lower electron transport chain coupling and greater hydrogen peroxide (H_2_O_2_) production were observed in mitochondria of low-FE birds [15]. A microarray gene expression analysis of breast muscle samples from high- and low-FE broiler chickens identified 782 differentially expressed genes [16,17]. Most of the genes up-regulated in high-FE chickens were related to anabolic metabolism, whereas genes up-regulated in low-FE chickens were associated with muscle fiber development, muscle function, cytoskeletal organization and stress response [16]. With the rapid development of next-generation sequencing technologies, RNA sequencing (RNA-seq) has been replacing microarray technology for transcriptome-wide gene expression analysis. Avoiding technical issues inherent to microarray such as cross-hybridization and narrow ranges of signal detection, RNA-seq can provide more accurate and comprehensive information regarding changes in gene expression between different conditions or different phenotypes [18-21]. Therefore, a global gene expression study using RNA-seq is required for better understanding the molecular basis of FE in broiler chickens.

The objective of this study is to characterize the biological basis of differences between high- and low-FE chickens through breast muscle RNA-seq analysis. Using tissue samples from extreme high- and low-FE broiler chickens, the present study identifies genes and pathways differentially regulated in breast muscle between these two groups of chickens, providing important information toward understanding the biological basis of variation in FE in broiler chickens.

## Methods

### Animals and sample collection

Six groups of 400 male commercial broiler chickens from a cross between three commercial broiler pure lines were sampled at 29 days of age from the field in the Delmarva region of the United States and transferred into individual cages for feed efficiency measurement. The cages were arranged in rows at two levels, top or bottom levels, and each row had 100 cages. The birds were individually weighed at the beginning (29 days of age, BW_29_) and end of the FE test (46 days of age, BW_46_) and fed *ad libitum* until 47 days of age. At 47 days of age, chickens were euthanized by cervical dislocation. Breast muscle samples (~1-2 g) were obtained from high- and low-FE birds, immediately flash frozen in liquid nitrogen and kept at -80°C until further processing. Body weight post-euthanization (BW_47_) and breast muscle weight (BMW_47_) were also recorded and used for estimating the percentage of breast muscle [(BMW_47_/BW_47_)*100]. The total feed consumption of each bird was measured by subtracting the total amount of feed left at the end of the test (46 days of age) from the total amount of feed provided to each bird at the beginning of the test (29 days of age). To measure the broiler’s FE, residual feed consumption (RFC) was calculated using the following equation:

$$ RFC = FC\ \hbox{--}\ \left( Level+ Row(Level) + b1*BW29 + b2*BW46 + c\right) $$

where FC represents the feed consumption of each bird; Level represents the fixed effects of row location (top or bottom level) on FC; Row (Level) represents the fixed effects of row nested within row location; BW_29_ is the initial (29-day) body weight; BW_46_ is the ending (46-day) body weight; c is the intercept; and b1 and b2 are the partial regression coefficients of FC on BW_29_ and BW_46_.

After excluding outliers and erroneous data (3.3%) and data from birds with defects (1.2%; leg and wings problem, etc.), samples from clinically healthy chickens exhibiting the highest (*n* = 12) and lowest (*n* = 13) RFC from the six groups of 400 birds were selected for cDNA library preparation. Two samples from the high-FE group did not produce enough cDNA libraries, so samples from 23 birds, 10 high- and 13 low-FE, were used for further analysis. The protocols were submitted to, and the use of the collected data and samples for research was approved by, the University of Delaware Agricultural Animal Care and Use Committee.

### RNA isolation

The frozen breast muscle samples were smashed into pieces by hammering. Pulverized tissues were stored at -80°C until RNA extraction. The total RNA was isolated from 70-100 mg of fragmented breast muscle tissues using a mirVana™ miRNA isolation kit (Ambion®; Austin, TX), according to the manufacturer’s protocol. RNA quantity and quality were assessed using a NanoDrop ND-1000 spectrophotometer (NanoDrop Technologies; Wilmington, DE) and Agilent 2100 bioanalyzer (Agilent Technologies; Santa Clara, CA). The RNA integrity numbers of all the RNA samples were above 8.0.

### RNA-seq library preparation and sequencing

In total, 23 cDNA libraries were constructed using an Illumina Truseq stranded RNA sample preparation kit following the manufacturer’s instruction (Illumina Inc.; San Diego, CA). Briefly, polyA containing mRNA molecules were purified by oligo (dT) magnetic beads and subsequently fragmented. The purified RNA fragments were reversely transcribed into first-strand cDNA using SuperScript II reverse transcriptase (Invitrogen™; Austin, TX). The second-strand cDNA was synthesized using dUTP instead of dTTP, as a result, the second-strand cDNA was not amplified during PCR because the polymerase can’t add nucleotide to dUTP. The double-strand cDNA was adenylated at the 3’ end and ligated to the Illumina indexing adapters. After PCR enrichment, cDNA quantity and quality were assessed using a NanoDrop ND-1000 spectrophotometer and Agilent 2100 bioanalyzer. The averaged size of synthesized cDNA fragments was approximately 260 bp. cDNA libraries were normalized to 10 nm for each sample and then pooled together and sequenced on four lanes of an Illumina Hiseq 2000 sequencer at Delaware biotechnology institute, University of Delaware. Approximate 68 million fragments per sample were sequenced by 75-bp from both ends.

### Mapping reads to the chicken reference genome

Before read alignment, the quality of raw sequence reads was checked using the FastQC program, and nucleotide calls with a quality score of 28 or higher were considered very good quality [22]. Sequencing reads from each sample were mapped to the chicken reference genome [Ensembl *Galgal4* (GCA_000002315.2)] using the TopHat program [23]. Because only the strand generated during the first-strand synthesis was sequenced, “-library-type fr-firststrand” was applied as one of the parameters in our read alignment using TopHat. Only one alignment for a given read was allowed in our analysis (i.e., -g 1), and both reads from a single sequence fragment were required to be mapped to the reference genome in a concordant manner (i.e., --no-discordant and --no-mixed). To summarize the alignments statistics, the resulting alignment files (SAM files) statistics were analyzed using SAMtools [24].

### Differential expression analysis

Cuffdiff, a companion tool of Cufflinks (v 2.1.1), was used to quantify the gene expression levels and to perform a differential expression test [25]. The fragment counts were normalized via a geometric method, as described previously [26]. Genes with a false discovery rate of less than 5% (i.e., FDR < 0.05) were considered significant.

### Nanostring nCounter® gene expression assay

The gene expression data was verified by NanoString nCounter® technology, as described previously [27]. Briefly, 23 RNA samples were submitted to NanoString, Inc. (Seattle, WA USA) for gene expression assay. With 12 housekeeping genes, 192 endogenous transcripts were selected across multiple on-going RNA-seq projects in our laboratory as target sequences to be measured. Designs of specific probes for target sequences were provided by NanoString [27] and were screened to avoid areas of high SNP density. A total of 100 ng of each RNA sample were hybridized to the CodeSet®, which was composed of both capture and reporter probes [27]. After 16 hours incubation, the samples were transferred to the nCounter® Prep Station and Digital Analyzer for transcript quantification. Positive control normalization factors and reference genes were used to normalize the raw data for biological analysis [27]. Log2 ratios of gene expression levels between high- and low-FE groups were calculated to compare with the corresponding log2 ratio values from RNA-seq analysis.

### Ingenuity pathway analysis

Genes differentially expressed (FDR < 0.05) between high- and low-FE birds were included in pathway and function analysis using Ingenuity pathway analysis (IPA; Ingenuity® Systems, http://www.ingenuity.com). The functional and canonical pathway analysis was used to identify the significant biological functions and pathways. Functions and pathways with *P*-value < 0.05 (Fischer’s exact test) were considered to be statistically significant. IPA’s upstream regulator analysis function was used to identify potential transcriptional regulators that could explain the observed changes in gene expression between high- and low-FE chickens. The activation z-score was calculated to predict activation or inhibition of transcriptional regulators based on published findings accessible through the Ingenuity knowledge base. Regulators with z-score greater than 2 or less than -2 were considered to be significantly activated or inhibited.

## Results and discussion

### Phenotype measurements

A summary of the phenotype measurements from 23 high-FE (*n* = 10) and low-FE (*n* = 13) chickens is presented in Table 1. Although the initial bird weights (BW_29_) are not significantly different between these two groups (*P* = 0.661), the mean body weight of high-FE birds is significantly heavier than that of low-FE birds at the end of the test (*P* < 0.05), and the high-FE chickens consumed significantly less feed than low-FE birds during the test (*P* < 0.01). Consequently, the difference in mean RFC values between high- and low-FE chickens is highly significant (*P* < 0.001). The mean breast muscle weight and breast muscle percentage of the high-FE birds are significantly higher than those of low-FE birds (*P* < 0.05).

**Table 1:** **Statistics of the measurements from high**- **and low**-**feed efficiency** (**FE**) **chickens**

**Measurements**	**High**-**FE birds** **(n** = **10)**	**Low**-**FE birds** **(n** = **13)**
Bird weight (Kg), 29-d	1.316 ± 0.140	1.345 ± 0.169
Bird weight (Kg), 46-d	3.093 ± 0.283	2.960 ± 0.176
Weight gain (Kg), 29- to 46-d	1.778 ± 0.188*	1.615 ± 0.099*
Feed consumption (Kg), 29- to 46-d	2.874 ± 0.249**	3.325 ± 0.136**
Feed conversion ratio^a^	1.620 ± 0.054**	2.063 ± 0.085**
Residual feed consumption (Kg)^b^	-0.276 ± 0.040**	0.356 ± 0.048**
Breast muscle weight (%BW), 47-d	23.2 ± 01.6*	21.6 ± 1.4*
Breast muscle weight (Kg), 47-d	0.721 ± 0.100*	0.648 ± 0.071*

The significance between high- and low-FE birds was determined using Fisher’s least significance difference (LSD) test. P ≤ 0.05 is denoted by *; P ≤ 0.01 is denoted by**.

^a^Feed conversion ratio = Feed consumption (29- to 46- d) / Weight gain (29- to 46-d).

^b^Residual feed consumption = FC – (Level + Row (Level) + b1*BW29 + b2*BW46 + c), where FC represents the feed consumption of each bird; Level represents the fixed effects of row location (top or bottom level) on FC; Row (Level) represents the fixed effects of row nested within row location; BW29 is the initial (29-day) body weight; BW46 is the ending (46-day) body weight; c is the intercept; and b1 and b2 are the partial regression coefficients of FC on BW29 and BW46.

### Transcriptional profile of chicken breast muscle

A total of 23 cDNA libraries were constructed using RNA samples of breast muscle tissues from 10 high- and 13 low-FE chickens and sequenced for 75 cycles from both ends on four lanes. In total, about 1.573 billion of 75-base sequence reads are obtained with an average of 393 million raw reads per lane. No significant difference in the number of reads between these four lanes is observed. The total number of reads for one sample ranges from 50 million to 88 million, with an average of 68 million reads per sample. Based on quality check reports, the averaged quality score of sequence reads is high, approximately 38, with the average GC content ranging from 49% to 51%. On average, 80% of the paired-end reads are properly mapped to the chicken reference genome (Ensembl *Galgal4*). The summary of alignment for all samples is shown in Additional file 1. The relative expression of a gene is normalized as fragments per kilobase of transcript per million mapped fragments (FPKM), which is proportional to the number of cDNA fragments originated from the gene transcript. The lowest limit of gene expression value is set to be 0.1 FPKM in at least one of the 23 samples. According to this limit, 14,148 genes are identified as being expressed in the breast muscle tissues. To assess the consistency of the gene expression levels between different samples, the Pearson’s correlation coefficient was calculated for each pairwise combination of samples [28]. The averaged pairwise correlation coefficient between samples is 0.794, reflecting pretty consistent gene expression profiles.

### Gene differential expression analysis

Differentially expressed genes were detected by Cuffdiff, an internal program of Cufflinks. Of 17,107 genes in the Ensemble database (Ensembl *Galgal4*), 1,059 were identified as significant genes with different expression levels between high- and low-FE chickens (q-value < 0.05) (Additional files 2 and 3). All of this group of 1,059 genes have a fold change greater than 1.3, and 642 genes (60.6%) have a fold change above 1.5. Among the 1,059 differentially expressed genes, 327 and 732 genes are down- and up-regulated in high-FE birds, respectively (Additional file 4). This relative imbalance in the number of down- and up-regulated genes is likely due to the increased breast muscle regeneration and inflammatory response in the high-FE chickens (discussed below). Since muscle development and inflammatory response require higher levels of activators such as growth factors, hormones and cytokines, the gene expression may be positively regulated by these activators in the breast muscle of the high-FE birds.

### Confirmation of RNA-seq data

**Figure 1:**
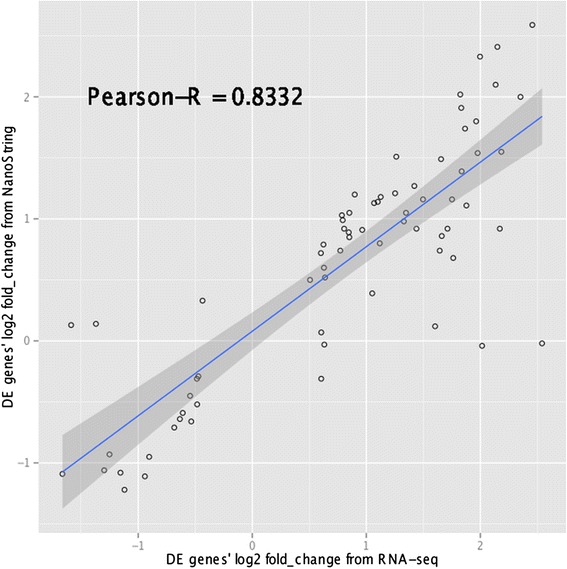
**Correlation of log2 fold**-**change between RNA**-**seq and NanoString for significantly differentially expressed target genes.** Pearson’s correlation of log2 fold-changes in gene expression levels, i.e. “log2 ratio (high-FE/low-FE)”, between results from RNA-seq and NanoString nCounter® technology for the 71 significantly differentially expressed target genes.

**Figure 2:**
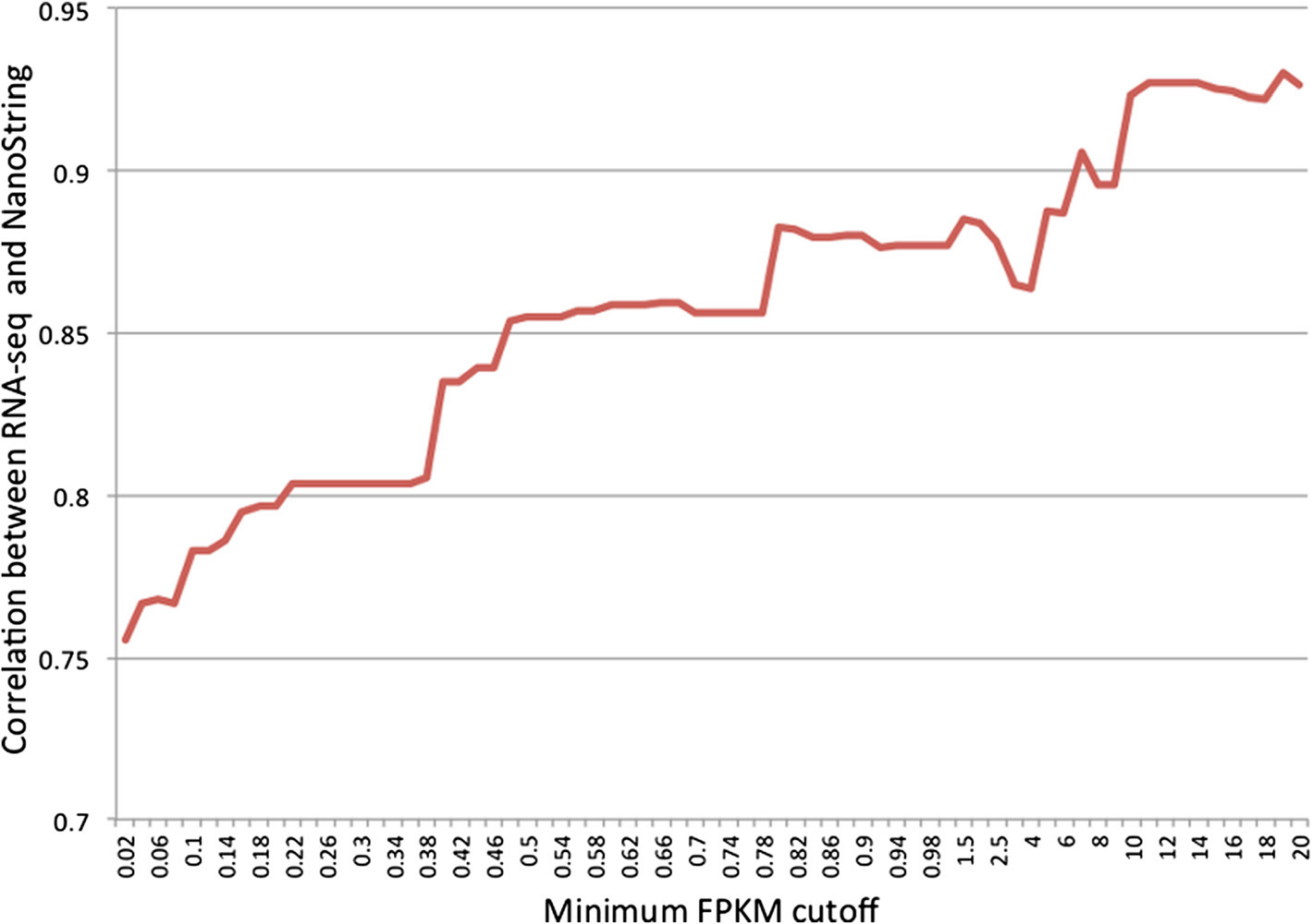
**The correlation of log2 fold**-**change between RNA**-**seq and NanoString increased with gene expression levels.** Pearson’s correlation of log2 fold-changes, i.e. “log2 ratio (high-FE/low-FE)”, between RNA-seq and NanoString is computed for different sets of target genes that are selected based on RNA-seq gene expression levels (FPKM value). X-axis represents the minimum FPKM cutoff used for gene filtering. Y-axis represents the Pearson’s correlation of log2 fold-change between RNA-seq and Nanostring.

To verify the gene expression data obtained from RNA-seq analysis, we selected 192 target genes (71 significant and 121 non-significant) along with 12 housekeeping genes for the NanoString nCounter® assay. Comparison of the normalized counts from NanoString with FPKM values derived from RNA-seq shows high concordance, with pair-wise Pearson’s correlation coefficients ranging from 0.70 to 0.98. The Pearson’s correlation coefficients of fold change in gene expression levels [log2 ratio(high-FE/low-FE)] between NanoString and RNA-seq results are also high: 0.7532 for all genes and 0.8332 for the 71 significantly differentially expressed genes (Figure 1). The correlation of log2 fold-change between two analyses is notably affected by lowly expressed genes, and increases by excluding genes with low expression levels (Figure 2). This can explain why the significantly differentially expressed genes show greater correlation compared with all the selected genes, because the FPKM values of the significant target genes are equal or greater than 0.4, whereas 23 genes out of the 121 non-significant target genes have an FPKM value less than 0.4.

### Overview of IPA analysis

To fully interpret the biological implications of the results from the differential expression analysis, all significant genes with their respective log2 fold-change were submitted for Ingenuity® Pathway Analysis. The top 10 up-regulated and top 10 down-regulated genes in high-FE chickens are listed in Additional file 5.

A summary of the IPA analysis, including top five biological functions and canonical pathways, are presented in Table 2. Generally, most of the differentially expressed genes are related to immune response and metabolic processes. Genes up-regulated in high-FE birds are associated with cellular function, movement, growth and proliferation, cell-to-cell signaling and interaction and cell death and survival. In contrast, genes down-regulated in high-FE birds are associated with metabolic processes including lipid, nucleic acid and carbohydrate metabolism, molecular transport and small molecule biochemistry (Table 3). Differing from the results of earlier work on chicken FE conducted using 44 K oligo microarray [16,17], genes involved in muscle fiber development, cytoskeletal organization and stress response are found to be up-regulated in the high-FE chickens (rather than in the low-FE chickens) in the current study. The discrepancy is probably from the different genetic composition of broiler chickens between two studies. Birds in previous studies are from a male broiler pure line that has been observed greater reactive oxygen species (ROS) production in the low-FE chickens than the high-FE birds [15], which is reverse of our findings that indicate ROS production is increased in the high-FE birds. Since ROS can act as a second messenger and mediate gene expression in the cell through signal transduction, differential gene expression is likely driven, in part, by the inherent differences that are modulated by ROS-mediated mechanisms. Further comparison of broiler chickens used in our study with pure line chickens used in these previous FE studies will be explained below.

**Table 2:** **Top biological functions and pathways enriched by differentially expressed genes between high**- **and low**-**FE chickens** ^**1**^

**Top molecular and cellular functions**		
**Functions**	**P**-**value**	**#Molecules**
Cellular movement	4.36E-18-2.57E-04	187
Cellular function and maintenance	2.38E-16-2.57E-04	241
Cell-to-cell signaling and interaction	1.23E-13-3.09E-04	167
Cellular growth and proliferation	2.89E-13-3.02E-04	259
Cell death and survival	1.70E-12-2.72E-04	256
**Top canonical pathways**		
**Pathways**	**P**-**value**	**Ratio**
Hepatic fibrosis/hepatic stellate cell activation	4.22E-09	23/146 (0.158)
Fcg receptor-mediated phagocytosis in macrophages and monocytes	1.65E-08	18/102 (0.176)
Leukocyte extravasation signaling	8.56E-07	24/207 (0.116)
Role of tissue factor in cancer	1.36E-06	17/116 (0.147)
PI3K signaling in B lymphocytes	9.94E-06	17/140 (0.121)

^**1**^A summary results from Ingenuity® Pathway Analysis (IPA) Software.

**Table 3:** **Top functions enriched by genes up**-**regulated or down**-**regulated in high**- **FE chickens**

	**Function annotation**	**P**-**value**	#**Molecules**
Genes down-regulated in high-FE chickens	Lipid metabolism	6.43E-06 -3.51E-02	34
	Molecular transport	6.43E-06 -3.54E-02	43
	Small molecule biochemistry	6.43E-06 -3.54E-02	62
	Nucleic acid metabolism	3.28E-05 -3.54E-02	21
	Carbohydrate metabolism	7.79E-05 -3.54E-02	35
Genes up-regulated in high-FE chickens	Cellular movement	3.78E-26 -7.96E-05	162
	Cellular function and maintenance	1.62E-19 -6.97E-05	194
	Cellular growth and proliferation	2.22E-19 -6.75E-05	210
	Cell-to-cell signaling and interaction	6.74E-18 -6.20E-05	145
	Cell death and survival	2.44E-15 -7.97E-05	198

Upstream regulator analysis through IPA predicted the cascade of upstream transcriptional regulators that can potentially explain the differences in gene expression profile between high- and low-FE chickens. A summary of the upstream regulators identified by IPA is presented in Additional file 6. A total of 27 transcriptional regulators are predicted to be activated or inhibited (24 activated and 3 inhibited) in the high-FE chickens, of which 24 regulators are considered to be significant with *P*-value < 0.05 (21 activated and 3 inhibited).

### Increased muscle growth and remodeling in high-FE chickens

Of all differentially expressed genes, 32 are associated with muscle development (Additional file 7), supporting the increased breast muscle weight in the high-FE birds. Among them, both *hepatocyte growth factor (HGF)* and *insulin-like growth factor 2 (IGF2)* encode key growth factors that have autocrine or paracrine effects on chicken skeletal muscle development and regeneration [29]. HGF can not only activate the proliferation of quiescent muscle satellite cells, it also can induce the migration of activated satellite cells to the injured sites [30]. IGF2 acts as a crucial regulator in muscle regeneration by stimulating muscle cell differentiation as well as inducing muscle cell hypertrophy [31]. Other muscle growth-related genes that are up-regulated in the high-FE chickens include *myogenin (MYOG), cysteine and glycine-rich protein 3 (CSRP3), myoferlin (MYOF), glypican 1 (GPC1), protein tyrosine phosphatase, receptor type, A (PTPRA)* and *gap junction protein (GJA1)*. As a member of myogenic regulatory factors (MRFs), MYOG is essential for the fusion of myoblasts into myotubes during muscle growth and regeneration [32]. The *CSRP3* gene encodes muscle LIM protein, which is able to increase the activity of MRFs and plays a critical role in enhancing myogenesis [33]. The *MYOF*-encoded protein is a fundamental modulator for myoblast fusion, highly expressed during muscle repair and regeneration [34]. The stimulatory effects of GPC1 on muscle satellite cell differentiation and myotube formation was reported in turkeys [35]. The protein encoded by the *PTPRA* gene is a signaling molecule that was found to increase myogenesis of rat muscle L6 cells [36]. The *GJA1*-encoded protein is a major component in gap junctions and required for myogenesis and regeneration [37]. Collectively, the up-regulation of genes that can positively regulate muscle growth indicates that muscle growth and development is elevated in the high-FE chickens.

In addition to genes involved in muscle development, genes associated with muscle hypertrophy, including *F-box protein 32 (FBXO32;* fold change = −1.879), *F-box protein 40* (*FBXO40*; fold change = −1.879), *F-box protein 9 (FBXO9*; fold change = −1.347), *forkhead box O3 (FOXO3*; fold change = −1.540) and *myotrophin* (*MTPN*; fold change = 1.426), are found differentially expressed in the breast muscle of chickens with high versus low FE. MTPN, a well-known positive growth factor in promoting muscle growth [38], is up-regulated in high-FE chickens. The increased MTPN expression may indicate that myocyte growth and protein synthesis are augmented in the breast muscle of high-FE birds, accordingly, contributing to breast muscle hypertrophy in these chickens. Furthermore, the down-regulation of FOXO3 and F-box family proteins in high-FE chickens further explains muscle growth differences between high- and low-FE chickens. Protein encoded by *FOXO3* is a master regulator of both autophagy-lysosome and ubiquitin-proteasomal pathways, promoting protein degradation and thereby contributing to muscle atrophy [39]. Proteins from the F-box family mediate the interaction between substrates and ubiquitin-conjugating enzymes, which facilitate proteolysis in diverse tissues [40]. Of them, the *FBXO32*-encoded protein, known as atrogin 1, is a well-recognized muscle-specific ubiquitin ligase leading to muscle atrophy in a wide range of diseases [41-43]. The *FBXO40*-encoded protein also has been proposed to play a role in muscle atrophy in mammals [44]. Thus, the decreased expression of atrophy-related genes in breast muscle of high-FE birds suggests that muscle protein loss is reduced in high-FE chickens in contrast to low-FE birds. The transcription of these genes is regulated by the PI3K/Akt signaling pathway, which will be discussed later. Taken together, the up-regulation of *MTPN* and down-regulation of *FOXO3* and FBXO family genes in the high-FE chickens suggest that birds with high FE may have elevated protein synthesis and decreased protein degradation in their breast muscle.

Genes associated with extracellular matrix (ECM) remodeling are also up-regulated in the high-FE birds. The ECM of skeletal muscle serves as a scaffold for maintaining the structure of muscle and guiding new fiber formation [45]. Muscle regeneration is frequently accompanied by the degradation of ECM because it facilitates satellite cell migration to specific sites for proliferation and fusion into myotubes [32,46]. Therefore, the up-regulation of genes involved in ECM remodeling implies that muscle remodeling is increased in the breast muscle of high-FE chickens. Matrix metalloproteinases (MMPs) are the main endopeptidases responsible for degrading all kinds of ECM, consequently, playing an important role in mediating muscle cell migration and regeneration [47,48]. As presented in Additional file 8, six genes from the MMP family are differentially expressed in our study, all of which are up-regulated in high-FE birds. Of the proteins encoded by these genes, MMP1 and MMP13 belong to MMP collagenases that are capable of cleaving interstitial collagen types I, II and III [49]. Through an *in vitro* wound-healing assay, MMP1 was able to promote myoblast migration and differentiation by increasing the expression of N-cadherin and β-catenin or pre-MMP-2 and TIMP [50]. MMP13 also plays a role in muscle regeneration, expressing in all muscle cells during muscle regeneration, and its expression level is positively correlated with the extent of muscle damage [51]. MMP9 is a gelatinase that also relates to muscle regeneration [49]. Evidence showed that the expression levels of MMP9 were greatly increased in response to inflammation and the activation of satellite cells in injured muscle [52-54]. However, contrary to the positive function of MMP1 on muscle regeneration, a recent study revealed that MMP9 could lead to muscle cell necrosis, inflammation and fibrosis [55]. Collectively, the up-regulation of MMPs in the high-FE birds suggests an augmented muscle remodeling in these birds compared with the low-FE chickens.

**Figure 3:**
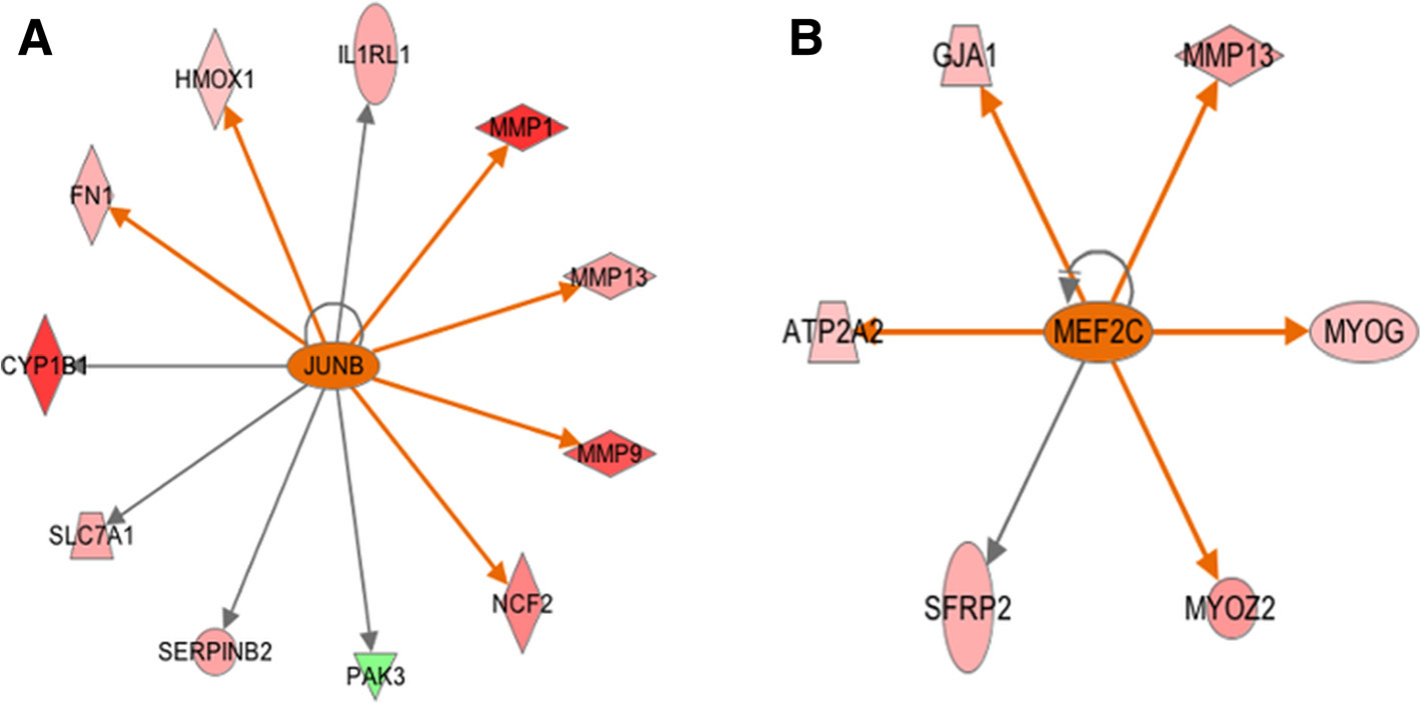
**Upstream regulators JunB and MEF2C. A**. Transcription factor JunB is predicted to be activated in the high-FE chickens by Ingenuity Upstream Regulator Analysis with *P*-value = 1.20E-03 and Z-score = 2.425. **B**. MEF2C is predicted to be activated in the high-FE chickens with *P*-value = 3.11E-02 and Z-score = 2.186. Red and green nodes indicate genes up- and down-regulated in the high-FE chicken, respectively. The color intensity is proportional to the degree of fold change. The upstream regulators are colored with their predicted activation states: orange (activated). Edges connecting the nodes are colored with orange when upstream regulators have activating effects on their target genes.

The IPA upstream regulator analysis provides additional support to our conclusions regarding muscle development and remodeling in the high-FE birds. According to the predictions from IPA, several transcriptional factors involved in muscle development are activated in high-FE chickens. As a regulator of postnatal muscle growth, JunB is predicted as being activated in the breast muscle of the high-FE birds (*P*-value = 1.2E-03, z-score = 2.200). JunB can stimulate myosin expression to elevate protein synthesis, accordingly, maintaining muscle mass and promoting muscle hypertrophy [56]. Additionally, JunB can suppress the transcription of *FOXO3* and thereby inhibit protein degradation in muscle [57]; therefore, activation of JunB in the high-FE birds may be one of the causes of the down-regulation of *FOXO3* as well as of *FBXO32* in breast muscle of these birds. In addition, through the IPA analysis, JunB is predicted to activate the transcription of *MMP1, MMP9, MMP13, fibronectin 1 (FN1), heme oxygenase 1 (HMOX1), neutrophil cytosolic factor 2 (NCF2) and interleukin 1 receptor-like 1 (IL1RL1)* (Figure 3A). As discussed above, FN1, MMP1 and MMP13 are all positively correlated with muscular satellite cell proliferation. Thus, JunB may also have increased myogenesis through activating transcription of these genes in the high-FE birds.

Apart from JunB, a main transcriptional factor in the formation of mature sarcomeres, myocyte-specific enhancer factor 2C (MEF2C), is predicted as being activated in breast muscle of high-FE birds [58,59]. Protein encoded by *MEF2C* is a member of the myocyte enhancer factor 2 (MEF2) family, which directly cooperates with MRFs and enhances skeletal muscle development [60]. In the present study, MEF2C is predicted to be an activated upstream regulator that increases the transcription of *GJA1*, *MMP13*, *MYOG*, *myozenin 2* (*MYOZ2*; fold change = 2.400) and *ATPase*, *Ca*++ *transporting*, *cardiac muscle*, *slow twitch 2 (*A*TP2A2)* (fold change = 1.390) (Figure 3B). GJA1, MMP13 and MYOG are all involved in myogenesis and exert positive effects on skeletal muscle growth and regeneration, which has been discussed above. Thus, MEF2C’s activation may augment the muscle development in high-FE birds. Moreover, *MYOZ2* is also predicted as being up-regulated by MEF2C. The *MYOZ2*-encoded protein belongs to a family of calcineurin-interacting proteins that modulates specific skeletal muscle signaling pathways through suppressing calcineurin [61]. It has been shown that MYOZ2 plays a role in regulating myocyte differentiation and promoting slow-oxidative fibers growth [62]. Collectively, MYOZ2 may be more active in breast muscle of high-FE chickens and, consequently, mediates some biological pathways and leads to muscle remodeling in these birds.

### Growth hormone (GH) and IGFs/PI3K/Akt signaling pathway over-represented in the differentially expressed genes

**Figure 4:**
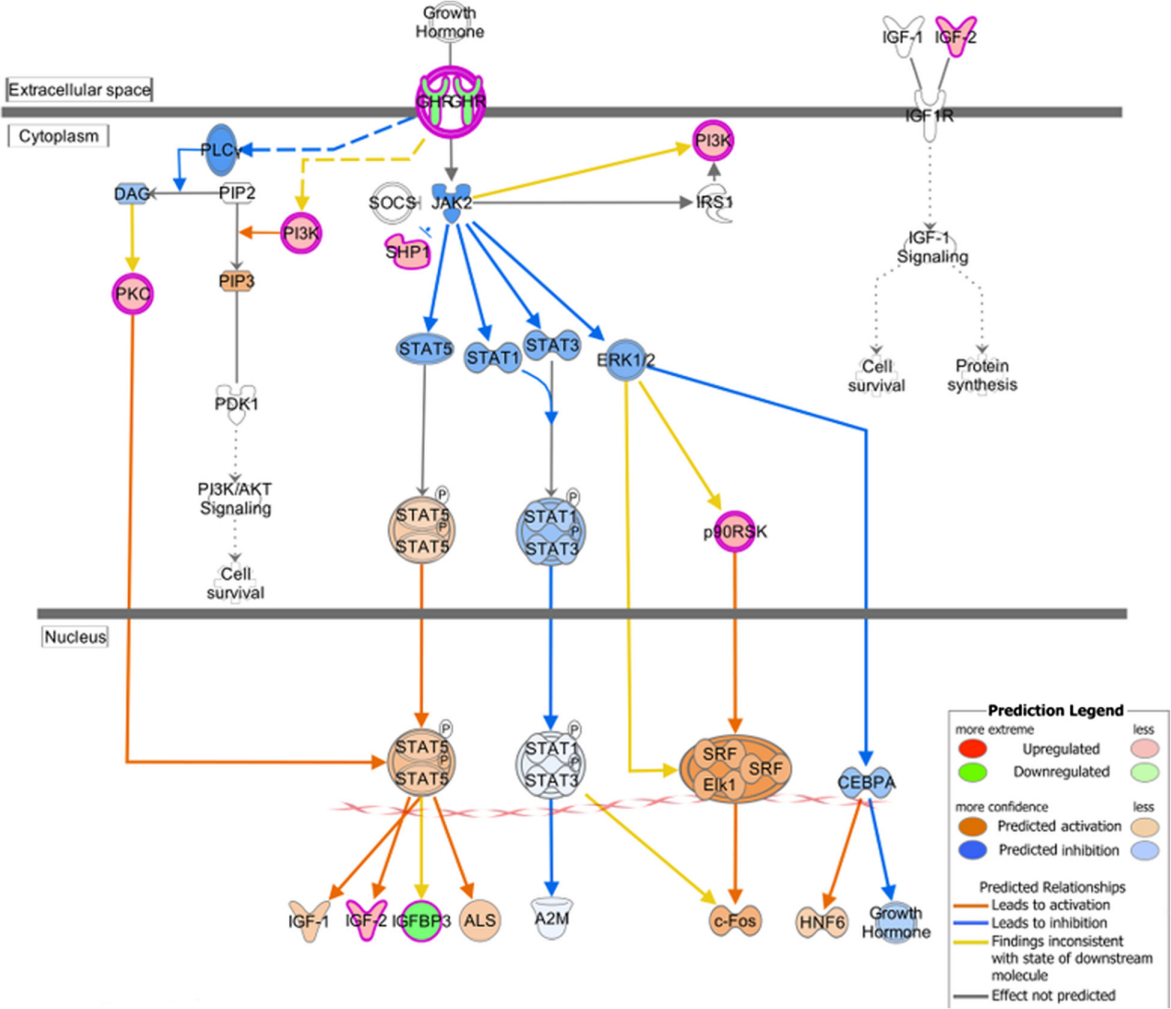
**Growth hormone signaling pathway analysis using Ingenuity Molecule Activity Predictor (MAP).** MAP predicts the upstream and downstream effects of the mapped genes on growth hormone signaling pathway and hypothesizes the overall states of this pathway. Red and green symbols indicate genes up- and down-regulated in high-FE chickens, respectively. Orange and blue nodes indicate genes predicted to be activated or inhibited in the high-FE chickens, respectively. The color intensity is proportional to the degree of fold change. Edges between the nodes are colored orange when leading to the activation of downstream genes, blue when inhibiting downstream genes. Yellow edges indicate that the states of downstream genes are inconsistent with the prediction based on previous findings.

Through the IPA canonical pathway analysis, several critical pathways in the regulation of body and muscle growth are over-represented among the differentially expressed genes. One of these pathways is the GH signaling pathway, enriched by 10 genes in our dataset (*P*-value = 3.25E-04; ratio = 1.32E-01) (Figure 4). As a key mediator of body size, GH has an anabolic effect on skeletal muscle development [63]. Through binding to growth hormone receptor (GHR) in muscles, biologically active GH can activate nuclear receptor STAT5 and thereby induce the synthesis and secretion of IGF-1 as well as IGF-2 (fold change = 1.657) [64]. Furthermore, GH can stimulate signaling molecules including PI3K [*phosphatidylinositol*-*4*,*5*-*bisphosphate 3*-*kinase*, *catalytic subunit beta* (*PIK3CB*; fold change = 1.663); *phosphatidylinositol*-*4*,*5*-*bisphosphate 3*-*kinase*, *catalytic subunit delta* (*PIK3CD*; fold change = 1.709); *phosphoinositide*-*3*-*kinase*, *regulatory subunit 5 (PIK3R5*; fold change = 1.564)] and protein kinase C (PKC) [*Protein kinase C delta type (PRKCD*; fold change = 1.558)], leading to the activation of Akt/PKB signaling pathway and STAT5 [65]. Both the PI3K/Akt/PKB pathway and IGFs are crucial contributors to muscle hypertrophy, which will be discussed later. Most of the mapped genes (8 of 10) are up-regulated in the high-FE chickens, indicating that the GH signaling pathway is more activated in the breast muscle of the high-FE birds compared with the low-FE birds. The two down-regulated genes [*GHR* and *insulin*-*like growth factor binding protein (IGFBP3*)] also fit this assumption. Evidence from the literature indicates that the expression of *GHR* is inversely correlated with the concentration of GH [66,67]. Thus, the down-regulation of *GHR* gene expression in the high-FE birds may be due to relatively high circulating GH levels in these birds. In spite of the stimulatory effects of GH on IGFBP3 transcription, there may be other modulators inhibiting the expression of *IGFBP3* in high-FE chickens, consequently exerting an inhibitory effect on IGF-1 function [68].

**Figure 5:**
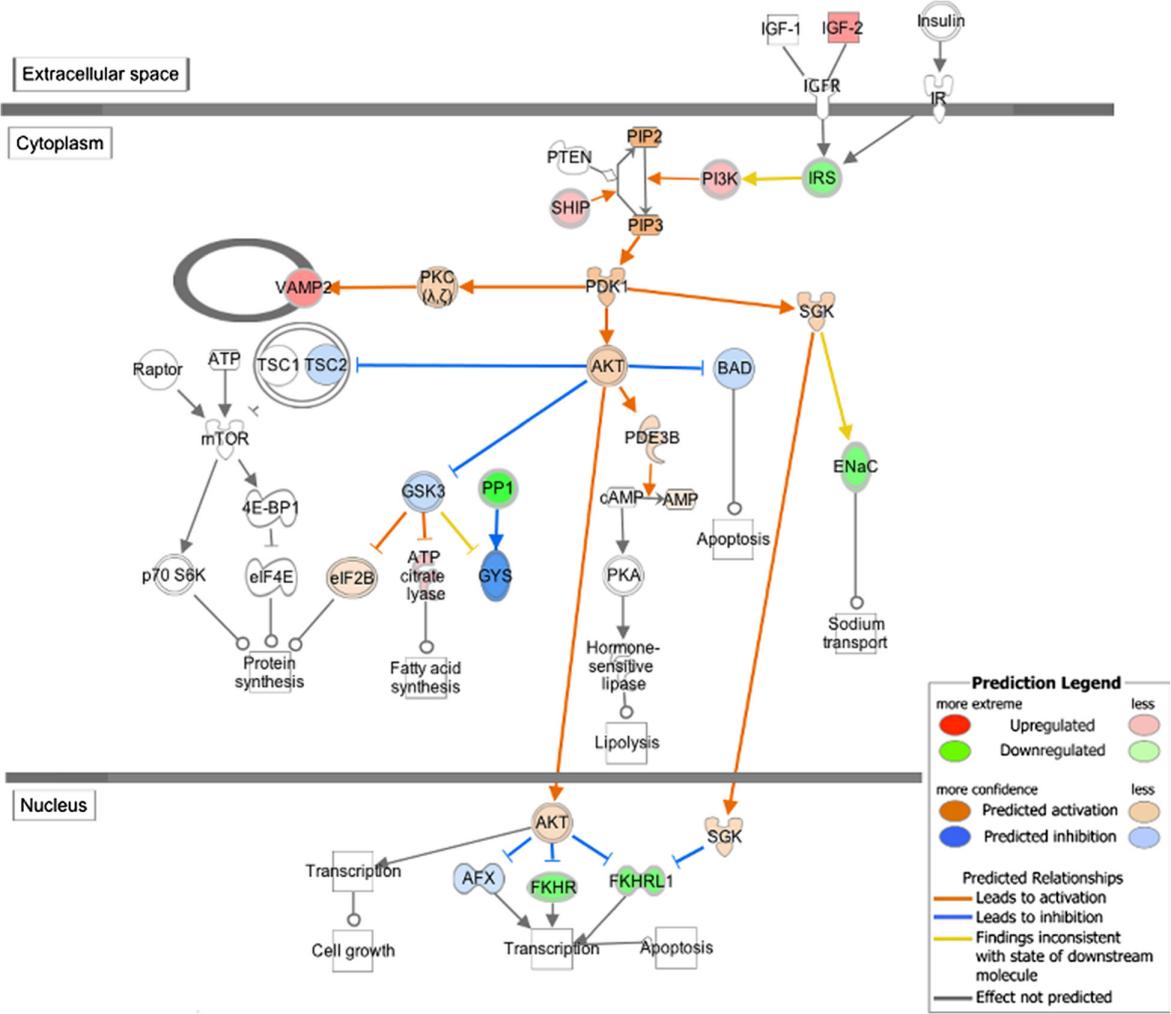
**IGFs**/**PI3K**/**Akt signaling pathway analysis using Ingenuity Molecule Activity Predictor (MAP).** MAP predicts the upstream and downstream effects of the mapped genes on IGFs/PI3K/Akt signaling pathway and hypothesizes the overall states of this pathway. Red and green symbols indicate genes up- and down-regulated in high-FE chickens, respectively. Orange and blue nodes indicate genes predicted to be activated or inhibited in the high-FE chickens, respectively. The color intensity is proportional to the degree of fold change. Edges between the nodes are colored orange when leading to the activation of downstream genes, blue when inhibiting downstream genes. Yellow edges indicate that the states of downstream genes are inconsistent with the prediction based on previous findings.

Another important finding is that the IGFs/PI3K/Akt signaling pathway is over-represented by the differentially expressed genes. The IGFs/PI3K/Akt signaling pathway plays a key role in the regulation of muscle growth and muscle hypertrophy in a variety of organisms [69-71]. Nine differentially expressed genes are mapped to the IGFs/PI3K/Akt pathway (Figure 5). Of these, *PIK3CD* (fold change = 1.709), *PIK3CB* (fold change = 1.663) and *PIK3R5* (fold change = 1.564) are up-regulated in the high-FE chickens, implying that the PI3K complex is more active in the breast muscle of these birds. The up-regulated members of the PI3K complex are predicted to increase PI3K-Akt cascade activity in the high-FE birds by IPA (Figure 5). Activated PI3K can induce the phosphorylation of phosphatidylinositol 4,5-bisphosphate (PIP2) to generate phosphatidylinositol-3,4,5-trisphosphate (PIP3). PIP3 acts as a docking site for phosphoinositide-dependent kinase 1 (PDK1) and Akt and subsequently contributes to the activation of Akt [72,73]. On the basis of the IPA prediction, the activated Akt translocates into the nucleus and then inhibits the transcription of the forkhead box O (FOXO) family, which is consistent with the down-regulation of the *forkhead box O3* (*FOXO3*) gene (fold change = −1.540) in our results [69]. As mentioned above, FOXO3 can promote protein degradation and muscle atrophy [39]. Hence, considering the expression profile of the mapped genes, the protein degradation process is predicted to be reduced in the breast muscle of the high-FE chickens as a result of PI3K/Akt pathway activation. In addition, because activation of Akt can up-regulate the transcription of *ATP citrate lyase (ACLY)* through suppressing the activity of glycogen synthase kinase (Gsk3), the increased expression of *ACLY* in the high-FE birds (fold change = 1.345) lends more support to the assumption that the PI3K/Akt pathway is activated in the high-FE chickens. Apart from repressing protein degradation, the activated PI3K/Akt pathway can promote protein synthesis in muscle via inhibiting Gsk3 [69], which is also inferred in our analysis. Therefore, the up-regulated IGFs/PI3K/Akt pathway suggests increased protein synthesis as well as decreased protein degradation in the breast muscle of the high-FE birds, explaining in part why high-FE chickens have more breast muscle than do low-FE birds. Previous studies on chicken FE also found that gene encoding PI3K was up-regulated in the high-FE chickens and a list of differentially expressed genes associated with the Akt/mTOR pathways, which strengthened our conclusion [17,74].

### Inflammatory response in the breast muscle of high-FE chickens

In the present study, a large number of the differentially expressed genes (136 genes) are involved in inflammatory response. Most of these genes (124 genes), including genes encoding for interleukin 8 (IL-8) and chemokine (C-X-C motif) ligand 14 (CXCL14), are expressed greater in the high-FE chickens. Although the cellular source of IL-8 and CXCL14 remains unknown in the current study, both not only exert direct effects on immune cell recruitment but also act as paracrine or endocrine factors in skeletal muscle. IL-8 has been recently classified as a myokine that can promote angiogenesis within the muscle [75,76]. CXCL14 is encoded by an obesity-induced gene in mice that inhibits the insulin-induced glucose uptake in cultured myocytes [77]. In addition, the gene encoding for corticotropin-releasing hormone (CRH) is also up-regulated in the high-FE birds (fold change = 2.824). Previous studies have demonstrated that CRH is secreted from nerve terminals and epithelial cells at inflammation sites and has a local proinflammatory effect on resident immune cells [78]. Therefore, it is likely that the elevated transcription of *CRH* functions to augment an immune response in the breast muscle of high-FE chickens. Apart from its immunomodulatory role, an increase in CRH may have a positive impact on thermogenesis of skeletal muscle in high-FE birds [79].

A series of genes encoding for cytokine receptors are also up-regulated in the high-FE chickens, further indicating an augmented immune response in the breast muscle of the high-FE chickens. These genes include *chemokine (C-C motif) receptor 2* (*CCR2*), *chemokine (C-C motif) receptor 5* (*CCR5*), *interleukin 17 receptor A* (*IL17RA*), *interleukin 18 receptor 1*(*IL18R1*), *interleukin 1 receptor, type I* (*IL1R1*), *interleukin 1 receptor*, *type II* (*IL1R2*), *interleukin 1 receptor*-*like 1* (*IL1RL1*), *interleukin 2 receptor*, *gamma* (*IL2RG*) and *interleukin 5 receptor*, *alpha* (*IL5RA*). Among them, *CCR2* was found to be expressed in infiltrating macrophages and playing a crucial role in muscle regeneration [80]. This gene can recruit macrophages to injured muscle, which then produces a high level of IGF-I to promote muscle regeneration [81]. Therefore, the up-regulation of *CCR2* suggests that, compared with the low-FE chickens, macrophage infiltration and muscle regeneration are increased in the breast muscle of the high-FE birds.

The IPA canonical pathway analysis also supports our hypothesis regarding augmented immune response and active recruitment of immune cells to the breast muscle of the high-FE chickens. Several over-represented pathways involved in inflammatory response are identified in our analysis (Additional file 9). Given that nearly all genes mapped to these pathways are up-regulated in the high-FE chickens, we conclude that these immune-related pathways are activated in the breast muscle of the high-FE chickens. According to the predictions from IPA, a number of transcription factors associated with inflammatory response are also activated in the breast muscle of the high-FE chickens: v-ets erythroblastosis virus E26 oncogene homolog 1 (ETS1), spleen focus forming virus (SFFV), spi-1 proto-oncogene (SPI1), X-box binding protein 1 (XBP1) and runt-related transcription factor 1(RUNX1).

**Figure 6:**
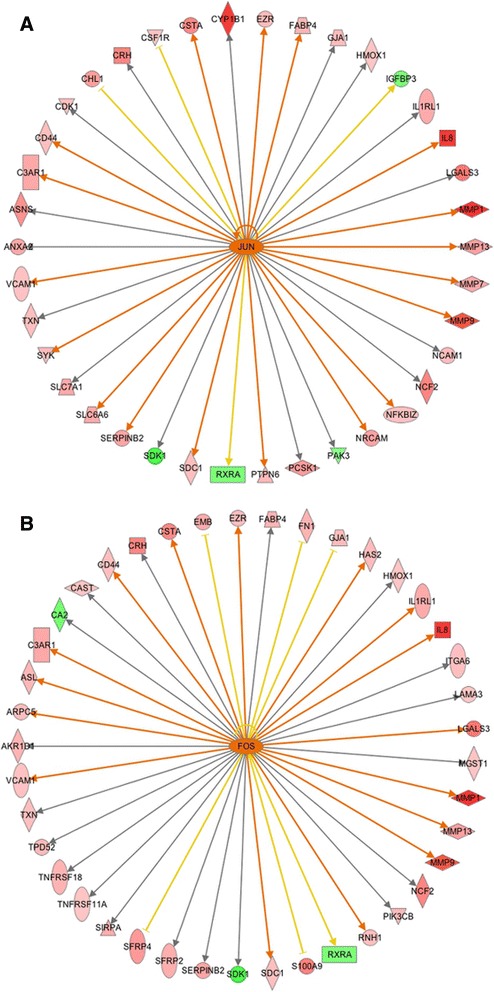
**Upstream regulator JUN and FOS. A**. Transcription factor JUN is predicted to be activated in the high-FE chickens with *P*-value = 1.70E-08 and Z-score = 2.923. **B**. FOS is predicted to be activated in the high-FE chickens with *P*-value = 7.64E-07 and Z-score = 2.277. Edges connecting the nodes are colored with orange when upstream regulators have activating effects on their target genes, blue when upstream regulators inhibit their downstream genes. Yellow edges indicate that the states of downstream genes are inconsistent with the prediction based on previous findings.

Muscle inflammation is a key step in muscle remodeling, which can clean disrupted muscle cells and promote muscle regeneration by releasing growth factors [82]. A variety of circumstances (e.g., muscle injury, exercise and obesity) can activate transcription factors NF-kB and c-Jun/FOS in muscle cells, resulting in the expression and secretion of several factors [83]. These factors, including cytokines and other non-protein mediators, can either directly attract circulating immune cells to the muscle or activate resident immune cells, providing chemotactic signals for recruitment [84]. As a consequence, a number of immune cells are recruited to the muscle, phagocytizing the cellular debris and producing cytokines affecting muscle cells [83,85]. The IPA upstream regulator analysis predicts that transcription factors JUN and FOS are activated in the breast muscle of the high-FE birds (Figure 6). Protein encoded by *JUN* and *FOS* are components of activator protein 1 (AP-1), which is an important transcription factor responding to various physiological and pathological stimuli [86]. Overall, our results suggest that, compared with the low-FE birds, the high-FE birds experienced a more intense muscle restructuring and higher inflammatory responses in the breast muscle.

### Free radical scavenging enriched in the differentially expressed genes between high- and low-FE chickens

Several differentially expressed genes in our dataset are involved in the production of ROS. Genes encoding for ROS-generating enzymes, including *cytochrome b*-*245*, *beta polypeptide* (*CYBB*) [fold-change = 2.08] and *NADPH oxidase organizer 1* (*NOXO1*) [fold-change = 2.38], are all up-regulated in the high-FE birds, suggesting that ROS production is increased in the breast muscle of these birds compared with the low-FE birds. CYBB, also known as NADPH oxidase 2 (NOX2), is a major enzyme responsible for superoxide production in the sarcoplasmic reticulum [87]. NOXO1, a positive mediator of NOX1 and NOX3, initiates the activity of NOX1 and NOX3 for generating ROS [88]. Moreover, the down-regulation of *uncoupling protein 3 (UCP3)* [fold-change = -1.67] in the high-FE birds may indicate that mitochondria from the breast muscle of the high-FE chickens have higher electron transport chain coupling compared with that from low-FE chickens. This assumption is consistent with previous findings [15,89]. Because UCP3-mediated uncoupling can attenuate the production of ROS [90], the down-regulation of *UCP3* in the high-FE birds may also suggest a higher production of ROS from the mitochondria of the breast muscle of these birds. Collectively, our data suggest that, compared with the low-FE birds, ROS is produced at a higher level in the breast muscle of the high-FE chickens.

**Figure 7:**
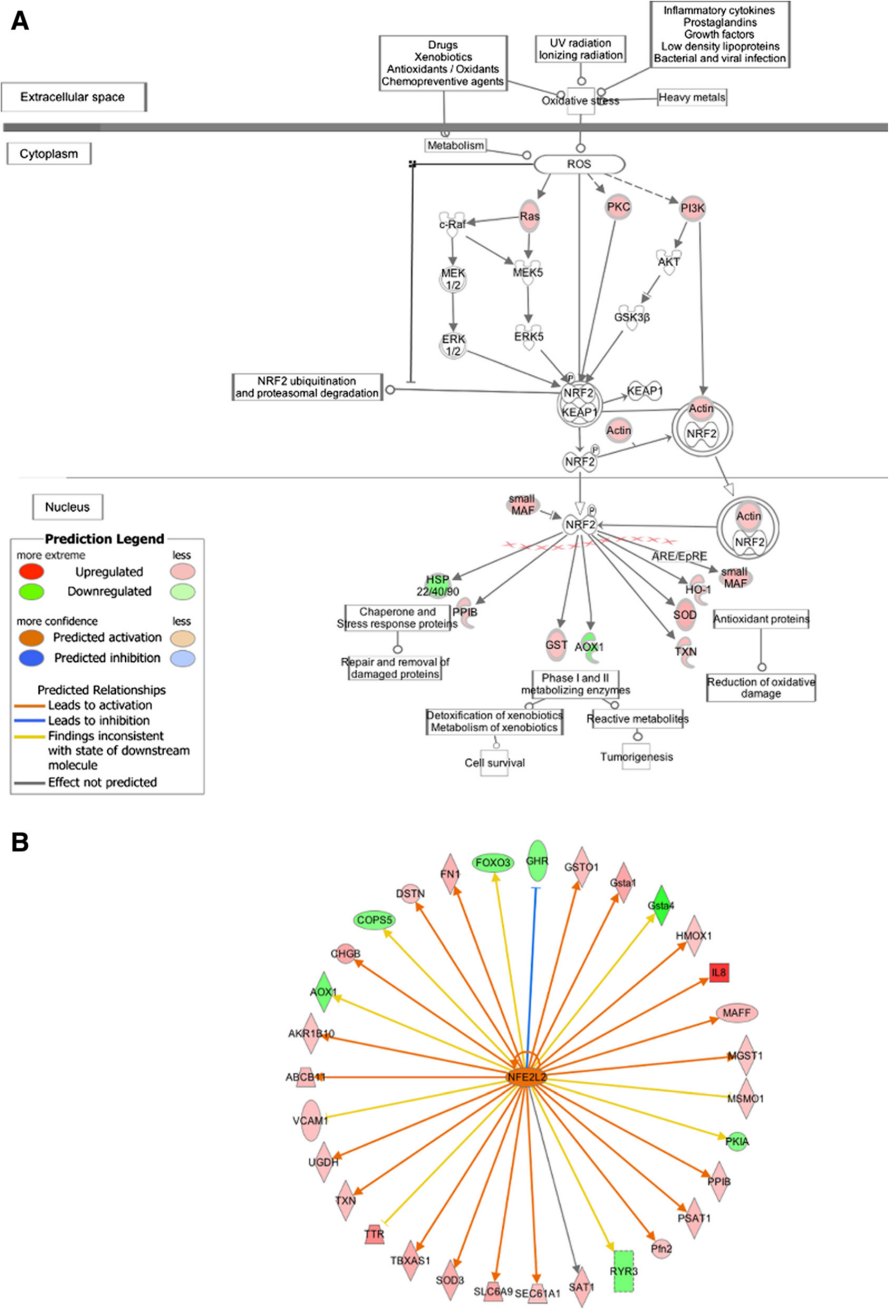
**NRF2**-**mediated oxidative stress response. A**. The Keap1-NRF2 pathway from IPA software. Canonical pathway analysis identified that the Keap1-NRF2 pathway was statistically significant with *P*-value = 6.96E-04. Red and green symbols indicate genes up- and down-regulated in the high-FE chickens, respectively. The color intensity is proportional to the degree of fold change. **B**. NRF2 (NFE2L2) is predicted to be activated in the high-FE chickens by Ingenuity Upstream Regulator Analysis.

The IPA downstream effect analysis supports our hypothesis regarding increased ROS production in the breast muscle of high FE-chickens. Processes, including metabolism of reactive oxygen species (*P*-value = 5.77E-07), synthesis of reactive oxygen species (*P*-value = 1.75E-06), production of reactive oxygen species (*P*-value = 4.92E-06) and production of superoxide (*P*-value = 2.05E-03), are predicted to be increased in the high-FE chickens. In addition, the NRF2-mediated oxidative stress response pathway is over-represented among the differentially expressed genes, with 17 genes (*P*-value = 6.96E-04; ratio = 0.089) mapped to this pathway (Figure 7A). Nuclear factor (erythroid-derived 2)-like 2 (NRF2), also known as NFE2L2, is a key transcription factor in cells that responds to a range of oxidative and xenobiotic stresses [91]. Upon exposure of cells to various stimuli such as ROS and electrophilic compounds, quiescent NRF2 in cytoplasm is activated through phosphatidylinositol 3-kinase (PI3K), RAS and PKC signaling pathways [92]. By phosphorylation or binding to actin, activated NRF2 translocates into the nucleus and binds to the antioxidant response elements, initiating the transcription of a number of genes encoding for antioxidants and ROS detoxifying enzymes [93]. A group of NRF2 downstream genes, including *v*-*maf musculoaponeurotic fibrosarcoma oncogene homolog F (avian)* (*MAFF*) [fold-change = 1.61], *glutathione S*-*transferase A3* (*GSTA3*) [fold-change = 2.03], *glutathione S-transferase omega 1 (GSTO1)* [fold-change = 1.50], *heme oxygenase (decycling) 1 (HMOX1)* [fold-change = 1.36], *microsomal glutathione S*-*transferase 1 (MGST1)* [fold-change = 1.39], *superoxide dismutase 3 (SOD3)* [fold-change = 1.85], *thioredoxin* (*TXN*) [fold-change = 1.50], *peptidylprolyl isomerase B* (*PPIB*) [fold-change = 1.43], *aldehyde oxidase 1* (*AOX1*) [fold-change = -1.63], *DnaJ (Hsp40) homolog, subfamily A*, *member 1* (*DNAJA1*) [fold-change = -1.39] and *DnaJ (Hsp40) homolog*, *subfamily C*, *member 1* (*DNAJC1*) [fold-change = -1.35], are differentially expressed in the current study. Genes encoding for antioxidant proteins, such as *SOD3*, *HMOX1* and *TXN*, are up-regulated in the high-FE birds. Protein encoded by *SOD3* is an extracellular protective enzyme against not only ROS but also inflammation, thus playing a role in tissue recovery [94]. HMOX1 is increased in the condition of oxidative stress and has an effect on protecting cells against ROS and inflammation [95]. The *TXN*-encoded protein is involved in a range of redox reactions and can decrease the quantity of ROS [96]. The up-regulation of *TXN*, *SOD3* and *HMOX1* indicates that an NRF2-mediated antioxidant response is activated in the breast muscle of the high-FE chickens. Additionally, three members from the glutathione s-transferase (GST) group, encoded by *GSTO1*, *GSTA1* and *MGST1*, are all up-regulated in the high-FE birds. GST is known for its function in detoxification of xenobiotics as well as endogenous metabolites [97]. The increased expression of the GST superfamily also suggests that responses to oxidative stress are elevated in the breast muscle of the high-FE chickens. Although few genes in the NRF2 signaling pathway, including *AOX1*, *DNAJA1* and *DNAJC1*, are down-regulated in the high-FE chickens, overall there are 14 up-regulated genes mapped to this pathway, indicating that NRF2-mediated oxidative stress response is augmented in the breast muscle of the high-FE birds. Moreover, NRF2 (NFE2L2), a transcription factor, is predicted to be activated in the high-FE chickens (*P*-value = 1.94E-05; z-score = 2.036) (Figure 7B). Taken together, our results suggest a higher level of ROS generated in the breast muscle of high-FE chickens.

However, in contrast to our findings, Bottje et al. (2002) reported higher amounts of ROS produced in the breast muscle of their low-FE birds [98]. This inconsistency is likely caused by the difference in broiler chickens between two studies. Male breeders, presumably with relatively low breast muscle yield, were studied in the Bottje et al. (2002) research [15], whereas we study broiler chickens from a commercial cross with high breast muscle yield. The ancestors of this cross have been intensively selected for the disproportionate growth of breast muscle, and the resulting higher levels of variation in breast muscle in the broiler cross may be responsible for a significant part of the variation in FE in this cross compared to the male breeder strain in the study by Bottje et al. [15]. In regard to broiler chickens in the current study, intensive inflammatory response is possibly a major source of increased ROS in the breast muscle of the high-FE chickens. ROS-generating enzymes, such as NOX in muscle cells, can be stimulated through extracellular inflammatory cytokines including interleukin (IL)-1, IL-6 and IL-8 in a ligand-induced pattern [99,100]. Furthermore, the implied infiltrating immune cells in the breast muscle of high-FE birds may be another cause for increased ROS. It is well known that immune cells produce a large amount of ROS to support their functions during inflammation [101]. Hence, in our study, strong indications for elevated ROS production in the breast muscle of the high-FE chickens are likely due to augmented inflammatory response, whereas the higher level of ROS observed in the study by Bottje et al. (2002) is possibly from mitochondria of breast muscle cells. Further study of genes associated with free radical scavenging may support our assumption. Indeed, in our study, a large part of these genes (Additional file 10) are also related to inflammatory response (*P*-value = 1.03E-23--5.15E-06), suggesting that production of ROS in the high-FE birds is closely associated with an increased immune response in the breast muscle.

Notably, growth factors including HGF, IGF-1 and fibroblast growth factor (FGF)-2 are also found to be able to induce intracellular generation of ROS in different types of cells [99]. As mentioned above, the breast muscle of high-FE birds have higher expression of *HGF* and *IGF*-*2*, which may play a role in stimulating ROS production in these birds. Moreover, such generated ROS exerts insulin-mimicking effects on the insulin/IGFs signaling pathway, which has shown to be a second messenger in insulin/IGFs signal transduction under physiological conditions [102]. Therefore, in the breast muscle of the high-FE birds, the insulin/IGFs receptor signaling pathway may be activated, in part, because of increased ROS production.

Higher ROS production may also lead to an increase in intracellular calcium concentration. It has been found that ROS mediates the influx of extracellular Ca^2+^ and mobilization of intracellular Ca^2+^ stores [103-105]. In the present study, genes involved in calcium transport [*solute carrier family 8*, *member B1* (*SLC8B1*), *phospholipase C*, *beta 2* (*PLCB2*) and *ATPase*, *Ca*^++^*transporting*, *cardiac muscle*, *slow twitch 2* (*ATP2A2*)] are all up-regulated in the high-FE birds, indicating increased calcium mobilization in the breast muscle of these birds. *ATP2A2* encodes sarcoplasmic reticulum Ca^2+^-ATPase isoform 2 (SERCA2), which is an important pump responsible for muscle relaxation through transporting Ca^2+^ from the cytosol into the sarcoplasmic reticulum lumen in muscle cells [106]. Because more SERCA2 are needed to maintain calcium homeostasis when high Ca^2+^ levels are present in cytosols, the up-regulation of *ATP2A2* in the high-FE birds may imply a high level of cytosolic Ca^2+^ in the breast muscle of these chickens compared with the low-FE birds.

### Transcriptional regulation of hypoxia-inducible factor-1α (HIF1α)

**Figure 8:**
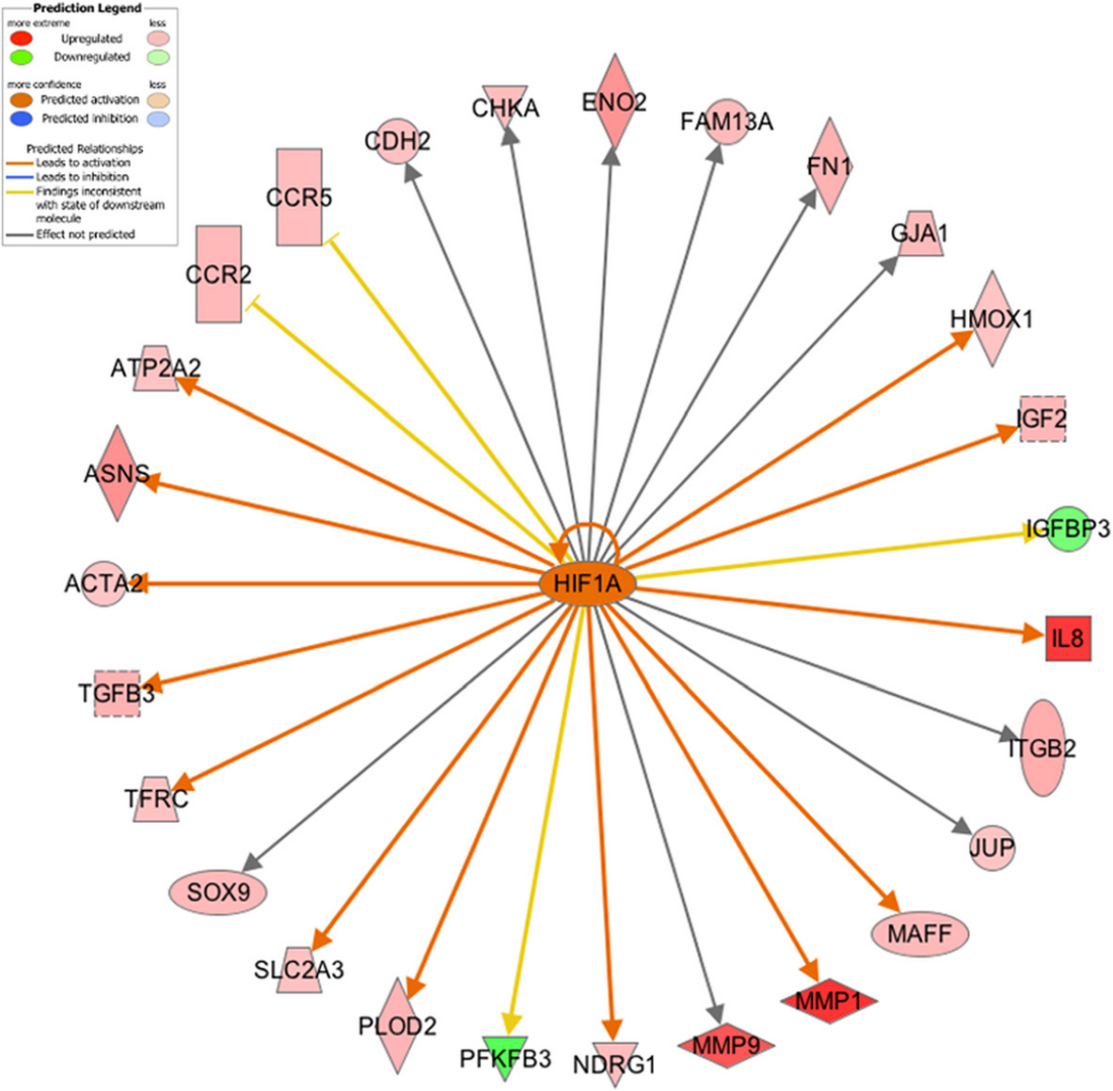
**Upstream regulator HIF1α and its target genes.** Transcription factor HIF1α is predicted to be activated in the high-FE chickens by Ingenuity Upstream Regulator Analysis.

Hypoxia-inducible factor-1α (HIF1α) is a key transcription factor that mediates cell adaption to hypoxia through regulation of a variety of gene expression [107]. Although *HIF1α* mRNA is constantly expressed in cells under both normoxic and hypoxic conditions, the HIF1α protein has a very short half-life in normoxia because of degradation through the ubiquitin-proteasome system [107]. During hypoxia, HIF1α degradation is repressed. As a result, HIF1α translocates into the nucleus and activates downstream genes in response to low O_2_ tension [107]. In our study, *HIF1α* mRNA, aryl-*hydrocarbon receptor nuclear translocator 2* (*HIF2β*) mRNA as well as *HIF1α* inhibitor *hypoxia inducible factor 1*, *alpha subunit inhibitor* (*HIF1AN*) mRNA are differentially expressed in the breast muscle between high- and low-FE chickens. *HIF1α* and *HIF2β* are up-regulated in the high-FE birds (fold change = 1.341 and 1.42, respectively), whereas *HIF1α* inhibitor *HIF1AN* is down-regulated in these birds (fold change = −1.343). Although the up-regulated *HIF1α* and *HIF2β* mRNA can’t represent increased amounts of stabilized HIF1α protein in the breast muscle of the high-FE chickens, decreased expression of *HIF1AN* may imply that HIF1α activity is increased in the breast muscle of the high-FE chickens compared with the low-FE birds. This assumption is supported by the expression of *HIF1α* downstream genes. As a transcription factor, HIF1α is predicted to be activated in the high-FE birds through the IPA upstream regulator analysis (*P*-value = 3.85E-06; z-score = 2.332; Figure 8). Indeed, a large number of the HIF1α target genes are up-regulated in the high-FE birds, indicating the activation of HIF1α in these birds.

Moreover, the HIF1α signaling pathway is over-represented among significantly differentially expressed genes (*P*-value = 7.58E-04; ratio = 1.39E-01). In response to hypoxia or a variety of peptide stimulators under normoxic conditions, PI3K/Akt and MAPK signaling pathways are activated to induce the accumulation of HIF1α in human cells [108,109]. Consequently, the accumulated HIF1α is translocated to the nucleus to modulate the transcription of genes involved in angiogenesis, glucose metabolism, matrix metabolism, erythropoiesis and apoptosis [107]. In our study, with increased expression of *PIK3CB*, *PIK3CD*, *PIK3R5 and muscle RAS oncogene homolog (MRAS)*, both the Akt/PI3K and MAPK signaling pathways are predicted to be activated in the high-FE chickens. The activated Akt/PI3K and MAPK signaling pathways may stimulate the induction of HIF1α, as reflected by the up-regulation of *HIF1α* and its downstream genes [*glucose transporter type 3* (*GLUT3*), *glucose transporter*-*like protein 5* (*GLUT5*), *matrix metallopeptidase 1* (*MMP1*), *matrix metallopeptidase 7* (*MMP7*), *matrix metallopeptidase 9* (*MMP9*), *matrix metallopeptidase 13* (*MMP13*), *matrix metallopeptidase 27* (*MMP27*), *matrix metallopeptidase 28* (*MMP28*) and *lactate dehydrogenase B* (*LDHB*)] in the high-FE birds. Based on the gene expression profile, we conclude that, compared with the low-FE birds, the activity of HIF1α signaling pathway is increased in the breast muscle of the high-FE birds.

Although it is unclear from our results whether hypoxia and/or mediators such as IGFs induced HIF1α activation in the breast muscle of the high-FE birds, we would like to speculate here about potential mechanisms underlying this activation. It is widely accepted that inflammation and hypoxia are closely interdependent in a wide array of physiological and pathological conditions [110-114]. Inflammation is frequently accompanied with hypoxia because of the high oxygen consumption of infiltrating immune cells [112]. Assuming an increased inflammatory response in the breast muscle of the high-FE birds, we speculate that the up-regulation of *HIF1α* is partly caused by an inflammation-induced hypoxia. Alternatively, the up-regulation of *HIF1α* may be caused by excessive muscle remodeling, which may be the result of selection for breast muscle proportion. Elevated muscle growth and rearrangements may have led to the reconstruction of vasculature, consequently reducing the blood flow and resulting in oxygen deficiency in the breast muscle of the high-FE birds [115]. Furthermore, insulin and IGFs have shown to be modulators of HIF1α induction during both normoxia and hypoxia [109]. Given that *IGF2* is up-regulated in the breast muscle of the high-FE birds, this growth factor may also have contributed to the activation of HIF1*α*.

Finally, the activation of HIF1*α* may also be partly due to a higher production of ROS in the breast muscle of the high-FE chickens. Studies have found that ROS are essential for the stabilization of HIF1-DNA, thereby triggering HIF1α-induced transcription [116,117]. It was also proposed that cellular ROS-producing proteins could sense changes in cellular oxygen concentration [118]. Evidence indicated that low oxygen tension inhibited mitochondrial electron transport and therefore increased ROS production. The generated ROS then acted as a second messenger that contributed to HIF1α activation [119]. Thus, the ROS production may have been increased in the breast muscle of the high-FE chickens partly because of a relatively low oxygen concentration within this tissue, which in turn may have played a role in HIF1α activation.

## Conclusions

The current study provides a global view of gene expression differences in the breast muscle of broiler chickens with extremely high and low FE from a population of a modern commercial high-meat-yield broiler cross. To our knowledge, this study reports for first time the RNA-seq analysis of a trait of selection and breeding importance in chickens. We identify 1,059 genes significantly differentially expressed in the breast muscle between high- and low-FE chickens based on the RNA-seq experiment. Furthermore, we achieve a large-scale validation of our RNA-seq experiment by quantifying the expression of a large number of target genes (192 transcripts + 12 house-keeping genes) using a high-sensitive non-PCR-based method, i.e. NanoString nCounter® Technology [27]. Function and pathway analysis of the differentially expressed genes sheds light on some of the underlying mechanisms that regulate chicken FE. Birds with high FE exhibit higher expression of genes involved in muscle growth, development and remodeling, which may explain why these birds have more breast muscle than do the low-FE chickens. Pathway analysis shows that anabolic pathways, including growth hormone signaling and IGFs/PI3K/Akt signaling pathways, are more activated in the high-FE birds, which may have not only led to the increased muscle growth in the high-FE chickens but also contributed to the feed conversion advantages of these birds. Our results also suggest that transcriptional factors JunB and MEF2C play crucial roles in regulating muscle growth and remodeling in high-FE chickens.

Furthermore, most of the genes up-regulated in the high-FE birds are associated with inflammatory response and oxidative stress, suggesting augmented inflammation and oxidative stress in the breast muscle of these birds. Our results also show increased activity of HIF1α, which may be caused by a lower oxygen environment in the breast muscle of high-FE chickens. Although no clinical symptoms of sickness or muscle damage were observed in the birds used in the current study, some of the molecular changes in the high-FE chickens may be hypothesized to lead to recently reported muscle quality issues in modern broiler chickens such as white striping and wooden breast [120-122]. These disorders have been reported to be more frequent in birds with high breast muscle weight and high FE, suggesting that the susceptibility may be primarily induced by breeding for these traits. Further investigation (e.g., histological and protein analysis) would be helpful for examining inflammation and oxidative stress in the breast muscle of high-FE and high-breast-muscle-yield birds.

## Availability of supporting data

The data supporting the results of this article are included within the article and its additional files. Readers may contact the corresponding author for additional information.

## Additional files

Additional file 1: Figure S1. The number of properly mapped, improperly mapped and unmapped reads is shown for each sample. Unmapped reads are reads not mapped onto the reference genome; Properly mapped reads are paired-end reads mapped to the reference genome and complying with the parameters (−g 1 –r 110 –no-discordant –no-mixed); Improperly mapped reads are reads mapped to the reference genome but not complying with the parameters. Additional file 2: Figure S2. Volcano plot showing differentially expressed genes between high- and low-FE chickens. Genes with q-value less than 0.05 were identified as significantly differentially expressed genes, which were shown in red, while genes with q-value larger than 0.05 were not statistically significant, shown in black. Additional file 3: Table S7. 1059 significant genes detected in the present study. Additional file 4: Figure S3. Comparison of gene expression between high- and low-FE broiler chickens. Genes up-regulated in the high- and low-FE chickens were marked with blue and red spots, respectively. Additional file 5: Table S1. Top 10 up-regulated and down-regulated genes in high-FE chickens. Additional file 6: Table S2. Activated or inhibited upstream transcription regulators predicted by Ingenuity® Pathway Analysis (IPA) Software. Additional file 7: Table S3. Differentially expressed genes involved in muscle development. Ingenuity® Pathway Analysis (IPA) Software identified 31 genes in our dataset that are associated with muscle development based on Ingenuity® Knowledge Base. Additional file 8: Table S4. Differentially expressed genes from matrix metalloproteinases (MMPs) family. Additional file 9: Table S5. Over-represented pathways involved in immune response. Additional file 10: Table S6. Differentially expressed genes associated with free radical scavenging are also involved in inflammatory response.
