# Supplementary figures and images for: Messenger RNA sequencing and pathway analysis provide novel insights into the biological basis of chickens’ feed efficiency
Source: BMC Genomics. 2015 Mar 17;16(1):195. doi: 10.1186/s12864-015-1364-0 (PMC4414306; doi:10.1186/s12864-015-1364-0)

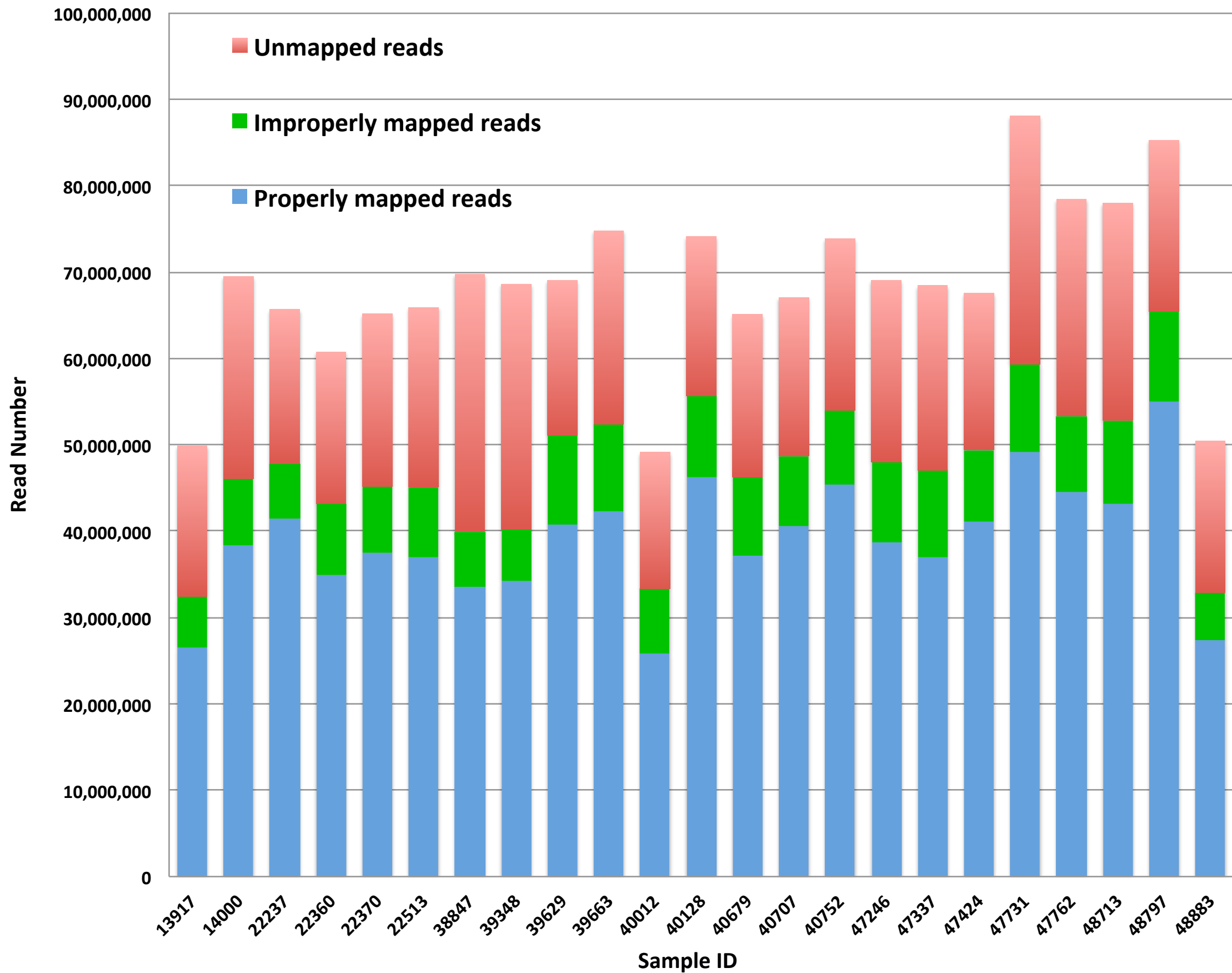

Supplement: Additional file 1: Figure S1. — The number of properly mapped, improperly mapped and unmapped reads is shown for each sample. Unmapped reads are reads not mapped onto the reference genome; Properly mapped reads are paired-end reads mapped to the reference genome and complying with the parameters (−g 1 –r 110 –no-discordant –no-mixed); Improperly mapped reads are reads mapped to the reference genome but not complying with the parameters. [file 12864_2015_1364_MOESM1_ESM.pdf]

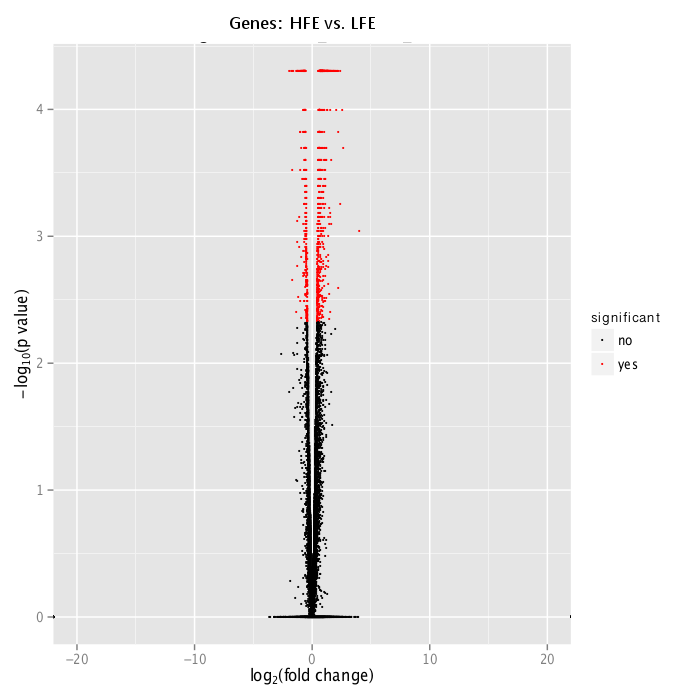

Supplement: Additional file 2: Figure S2. — Volcano plot showing differentially expressed genes between high- and low-FE chickens. Genes with q-value less than 0.05 were identified as significantly differentially expressed genes, which were shown in red, while genes with q-value larger than 0.05 were not statistically significant, shown in black. [file 12864_2015_1364_MOESM2_ESM.png]

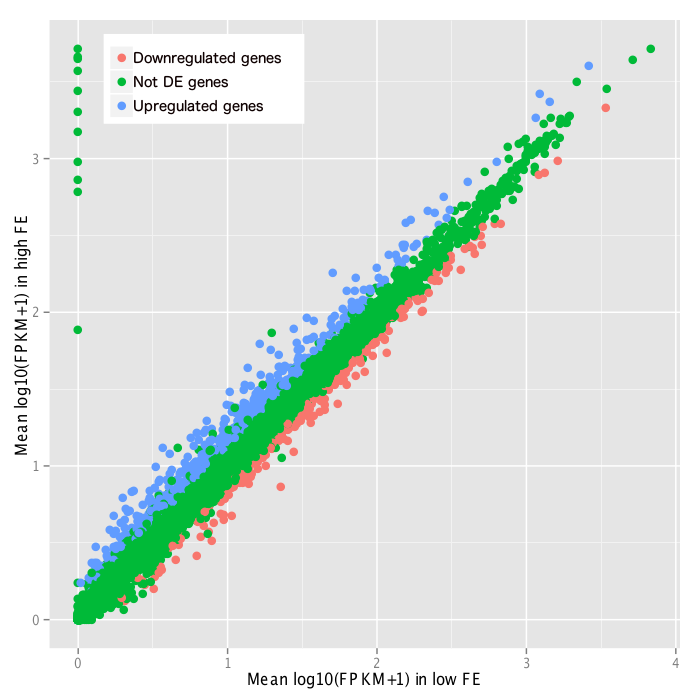

Supplement: Additional file 4: Figure S3. — Comparison of gene expression between high- and low-FE broiler chickens. Genes up-regulated in the high- and low-FE chickens were marked with blue and red spots, respectively. [file 12864_2015_1364_MOESM4_ESM.png]

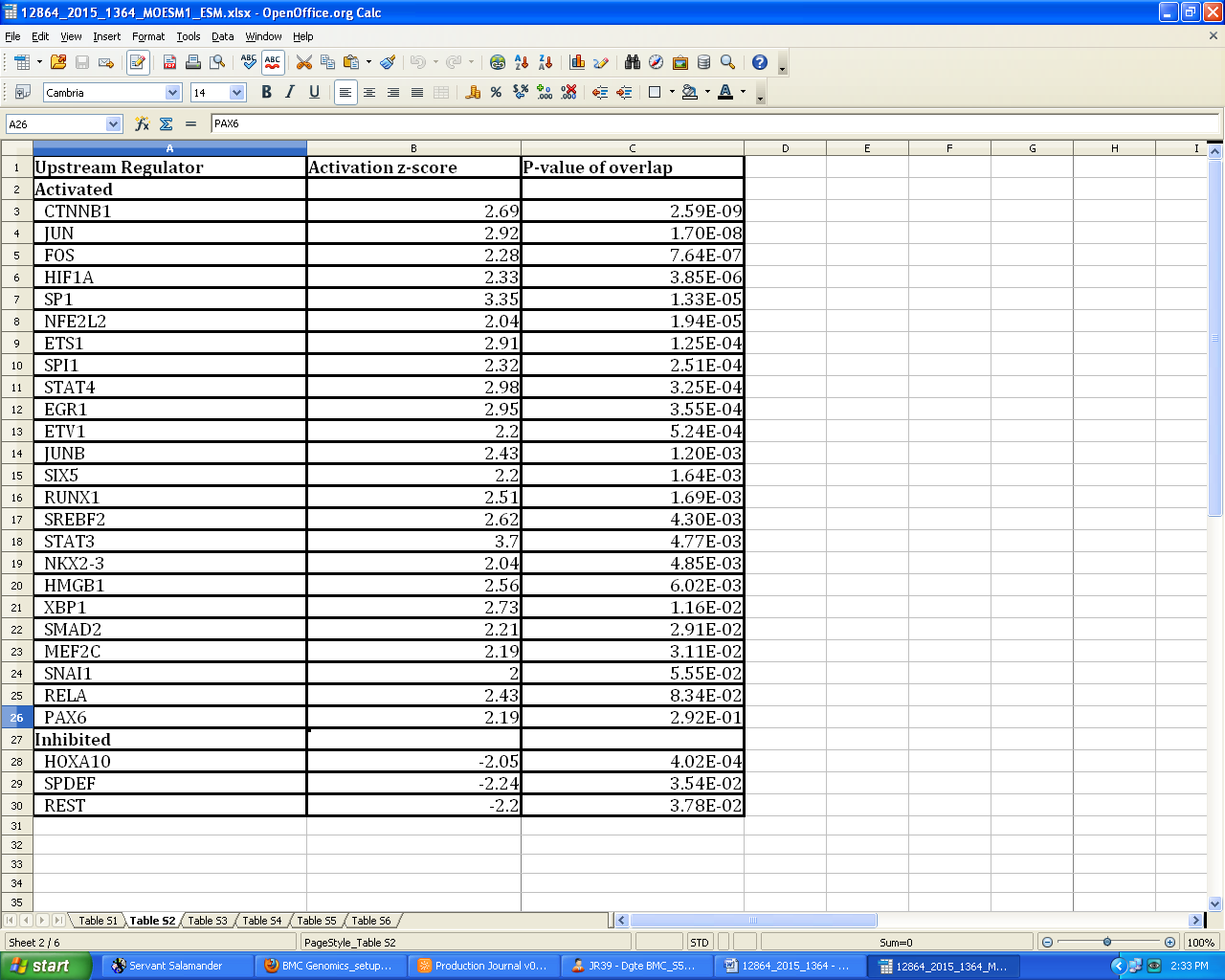

Supplement: Additional file 6: Table S2. — Activated or inhibited upstream transcription regulators predicted by Ingenuity® Pathway Analysis (IPA) Software. [file 12864_2015_1364_MOESM6_ESM.doc]

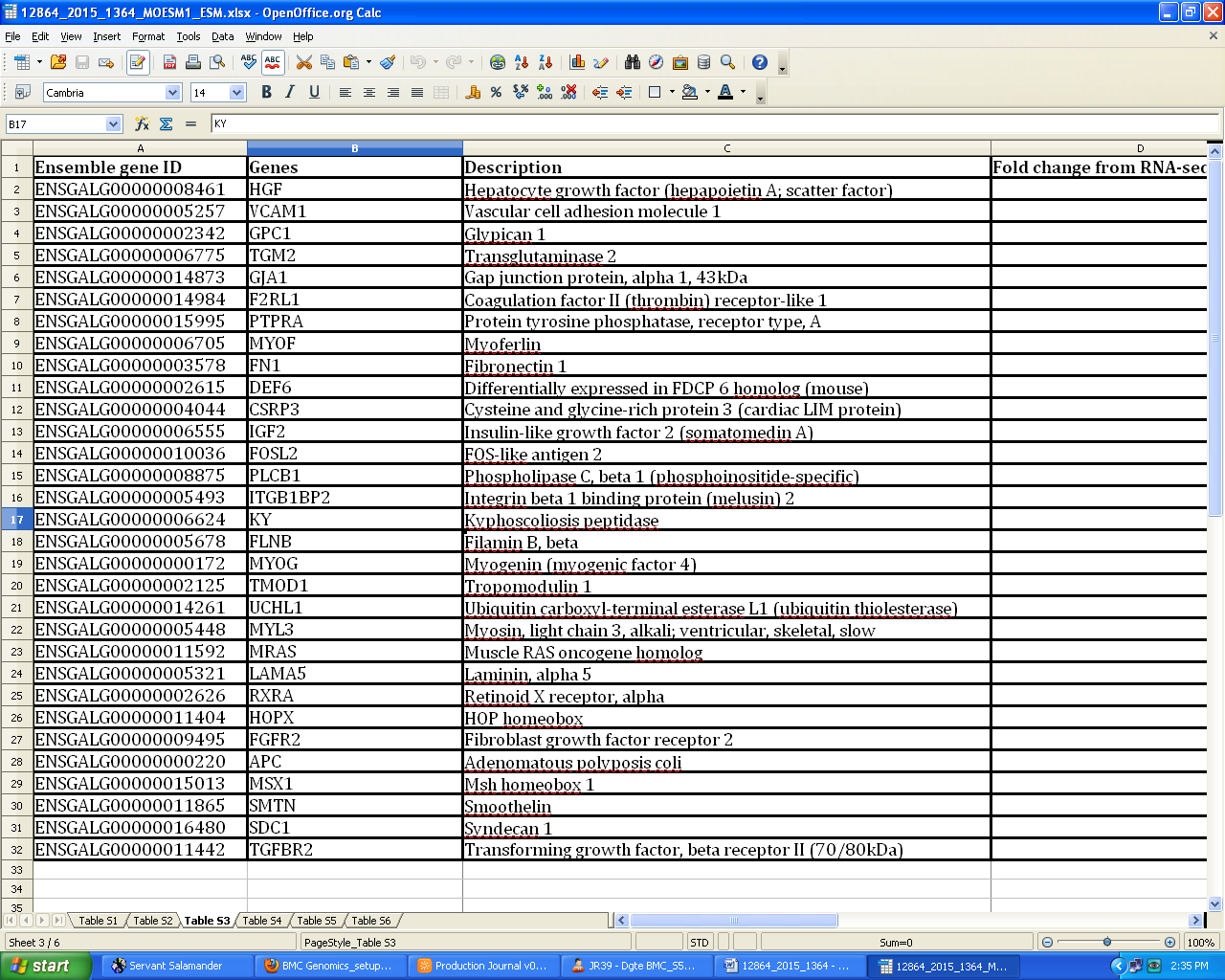

Supplement: Additional file 7: Table S3. — Differentially expressed genes involved in muscle development. Ingenuity® Pathway Analysis (IPA) Software identified 31 genes in our dataset that are associated with muscle development based on Ingenuity® Knowledge Base. [file 12864_2015_1364_MOESM7_ESM.doc]

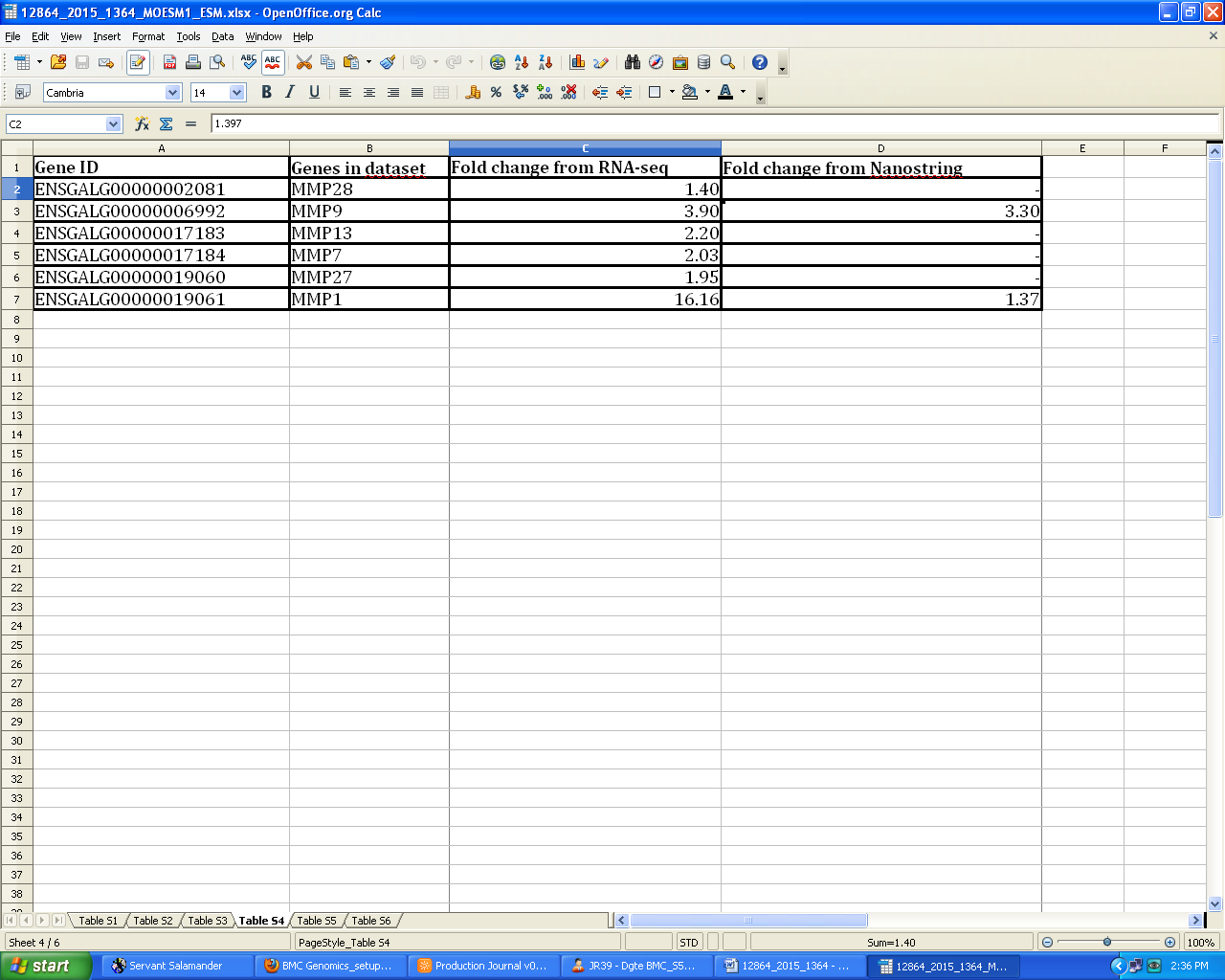

Supplement: Additional file 8: Table S4. — Differentially expressed genes from matrix metalloproteinases (MMPs) family. [file 12864_2015_1364_MOESM8_ESM.doc]

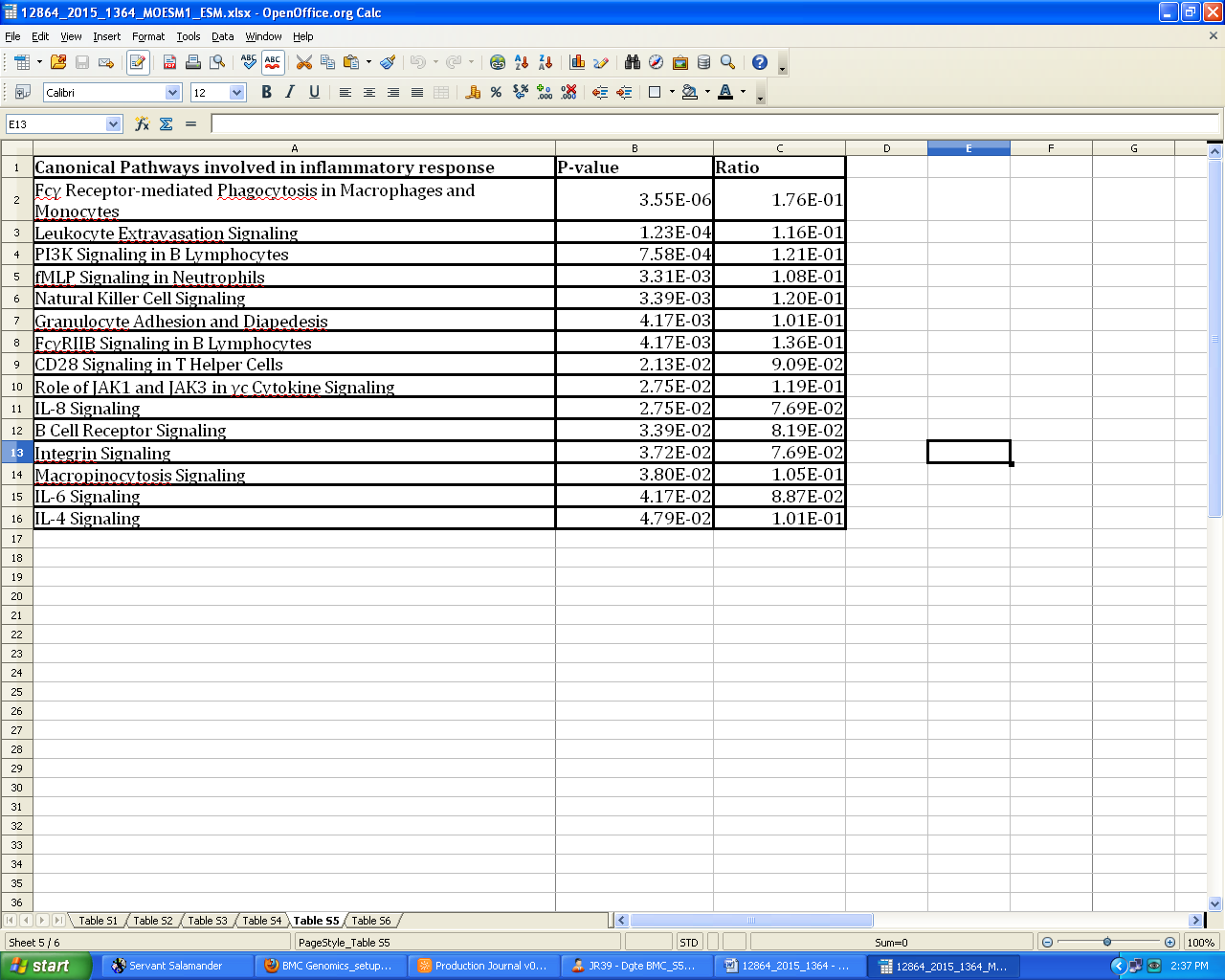

Supplement: Additional file 9: Table S5. — Over-represented pathways involved in immune response. [file 12864_2015_1364_MOESM9_ESM.doc]

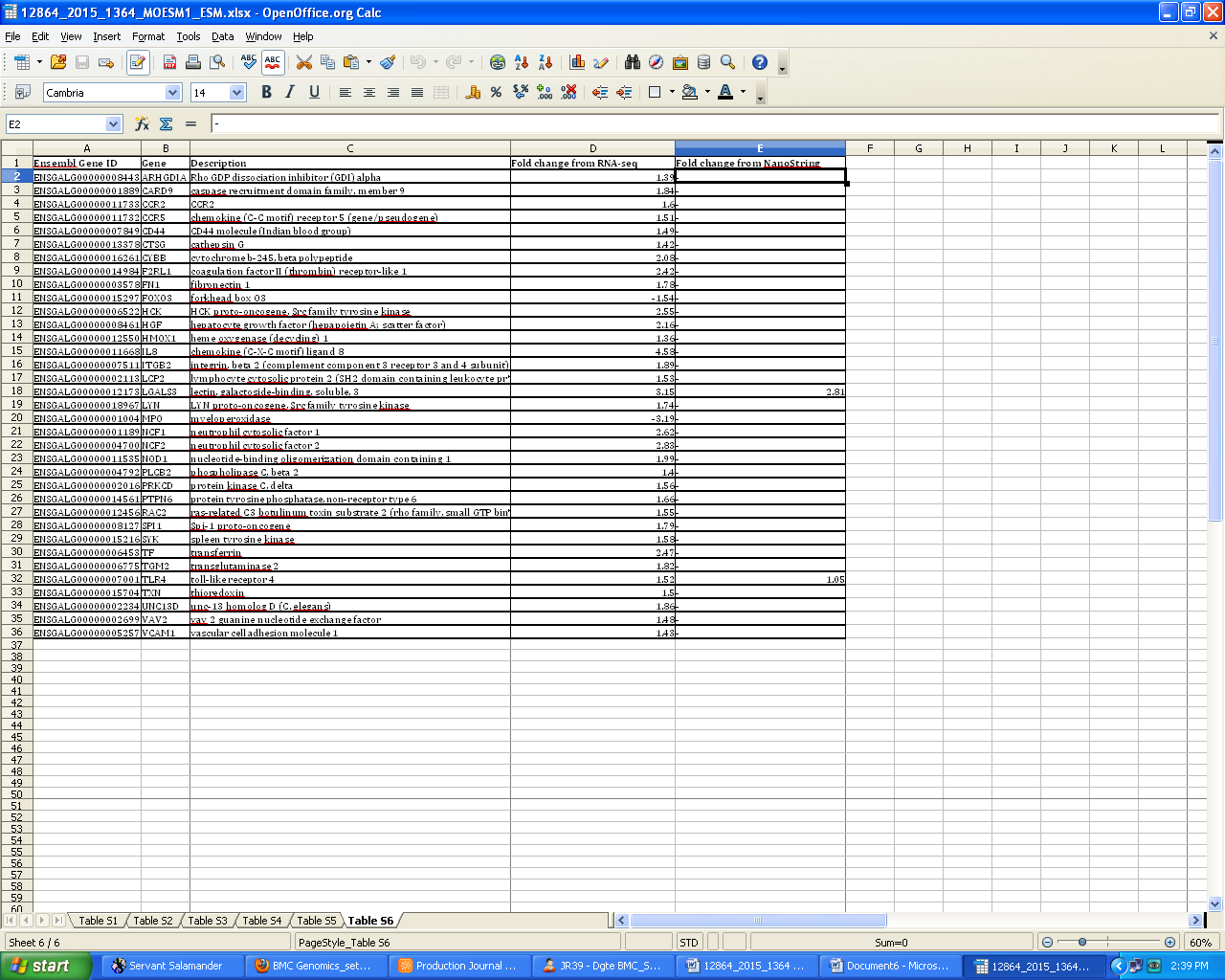

Supplement: Additional file 10: Table S6. — Differentially expressed genes associated with free radical scavenging are also involved in inflammatory response. [file 12864_2015_1364_MOESM10_ESM.doc]
